# Safety and efficacy of East Asian herbal medicine for iron deficiency anemia in children and adolescents: a systematic review and meta-analysis

**DOI:** 10.3389/fphar.2024.1339486

**Published:** 2024-04-08

**Authors:** Yoon Kyoung Jeong, Jae Hyun Kim, Sun Haeng Lee, Miran Bang, Gyu Tae Chang

**Affiliations:** ^1^ Department of Korean Pediatrics, College of Korean Medicine, Kyung Hee University, Kyung Hee University Medical Center, Seoul, Republic of Korea; ^2^ Jeongseon Public Health Center, Gangwon-do, Republic of Korea; ^3^ Department of Korean Pediatrics, College of Korean Medicine, Kyung Hee University, Kyung Hee University Hospital at Gangdong, Seoul, Republic of Korea

**Keywords:** iron deficiency anemia, children, treatment, herbal medicine, systematic review, meta-analysis

## Abstract

**Background:**

This systematic review and meta-analysis aimed to evaluate the efficacy and safety of East Asian herbal medicine (HM) for iron deficiency anemia (IDA) in children and adolescents.

**Methods:**

Twelve electronic databases were searched in 28 May 2023 for randomized controlled trials (RCTs) evaluating the efficacy of HM in children with IDA. The primary outcome measures for treatment included blood hemoglobin and serum ferritin levels, whereas the secondary outcomes included the total effective rate (TER), incidence of adverse events (AEs), average healing time, and hematologic indicators related to IDA. Meta-analysis was performed using Review Manager 5.4 and R studio 4.3 software, and subgroup analyses were performed according to the different groups (type of intervention and duration of treatment). The effect size measures used were the risk ratio, mean difference, and standardized mean difference with 95% confidence intervals. The risk of bias was assessed using a revised Cochrane risk of bias tool for randomized trials, and the quality of evidence was assessed using the Grading of Recommendations Assessment, Development, and Evaluation tools.

**Results:**

The meta-analysis included 28 studies. Five studies compared the efficacy of HM alone with that of oral iron alone, whereas 23 compared the efficacy of a combination of HM and oral iron with that of oral iron alone. The meta-analysis revealed that the HM treatment group showed significant improvements in all outcome measures compared to those observed in the oral iron group. Moreover, HM significantly reduced the incidence of gastrointestinal AE, compared to that associated with oral iron. Among the 28 studies, the most commonly used HM prescription was Xingpi Yanger Granules, and the most commonly used botanical drug was *Astragali Radix*, followed by *Atractylodis Rhizoma Alba* and *Angelicae Sinensis Radix*.

**Conclusion:**

This meta-analysis identified evidence of the efficacy and safety of HM in children with IDA. Compared to conventional oral iron therapy, HM improved IDA-related blood markers and TER, with fewer AEs and shorter mean healing times. However, further well-designed, large-scale clinical trials are necessary to strengthen the evidence of the efficacy and safety of botanical drugs.

**Systematic Review Registration:**

PROSPERO 2022 CRD42022334670. (https://www.crd.york.ac.uk/prospero/display_record.php?ID=CRD42022334670).

## 1 Introduction

Anemia is prevalent in infants and children; iron deficiency anemia (IDA) is the most common type of anemia in children (Wang, 2016). According to the World Health Organization, approximately 42% of preschool children <5 years old have anemia globally (Ning and Zeller, 2019; Belali, 2022), and IDA is estimated to be present in 20%–25% of children in this age group (Gedfie et al., 2022). Anemia is defined as hemoglobin (Hb) levels of less than two standard deviations of the mean age (Wang, 2016), and laboratory findings of IDA include low serum iron (SI), serum ferritin (SF), mean corpuscular volume (MCV), and mean corpuscular hemoglobin (MCH), transferrin saturation (TS), and high total iron binding capacity (TIBC) (Johnson-Wimbley and Graham, 2011).

Although IDA may be asymptomatic or present a few mild symptoms, such as fatigue, if left untreated, severe cases can have long-term effects on children’s development, such as neurodevelopmental and cognitive disorders (Subramaniam and Girish, 2015). Additionally, IDA can increase susceptibility to infection by affecting immune cell proliferation (Subramaniam and Girish, 2015; Hassan et al., 2016); therefore, early treatment is an important topic in clinical research.

Oral iron therapy is typically the first-line treatment for children with IDA (Ning and Zeller, 2019). However, it has several limitations such as poor body absorption, low bioavailability, and frequent gastrointestinal side effects, including abdominal discomfort, constipation, nausea, vomiting, and dark-colored stools (Johnson-Wimbley and Graham, 2011; Cappellini et al., 2020). Additionally, continuous treatment is required for at least 3 months after anemia is corrected, which can lead to poor compliance in children and act as a barrier to successful oral iron therapy (Powers et al., 2016). Recent studies have shown that the administration of herbal medicines (HMs) to children with IDA effectively improves IDA-related hematological markers (Li et al., 2022; Zhang et al., 2022). Two systematic reviews have reported on the treatment of IDA with botanical drugs (Li et al., 2022; Zhang et al., 2022); however, neither focused on children, but on pregnant women or on the entire population. Furthermore, they only analyzed the therapeutic effects of Shengxuening tablets, which were known to remove blood stasis, replenish qi and nourish blood (Li et al., 2022; Zhang et al., 2022). Since there has been no systematic review evaluating the overall efficacy of HM focusing on children, this meta-analysis aimed to evaluate the safety and efficacy of East Asian botanical drugs for IDA in children and adolescents.

## 2 Methods

### 2.1 Eligibility criteria

#### 2.1.1 Type of research

Only randomized controlled trials (RCTs) comparing the efficacy and safety of HMs with oral iron therapy in children and adolescents with IDA were included. All the studies used a randomized assignment methodology and were not restricted by language, blinding, publication year, or publication status. Studies with incomplete data, duplicate publications, animal studies, theses, meta-analyses, and studies involving other traditional Chinese medicine (TCM) interventions were excluded. Because this was a systematic review and secondary analysis of previous studies, neither ethical approval nor patient-informed consent were required.

#### 2.1.2 Participants

Children aged <18 years diagnosed with IDA according to clear diagnostic criteria were included regardless of nationality, sex, race, or disease duration. Patients with infectious diseases, congenital heart diseases, or other serious underlying conditions were excluded. Patients who had received iron supplementation before enrollment were also excluded.

#### 2.1.3 Interventions and comparisons

Studies on East Asian HMs were included regardless of the type of preparation (e.g., powders, granules, decoctions, solutions, capsules, or pills), intervention HM type (e.g., fixed, individualized, patent proprietary), and treatment duration. TCM interventions except botanical drugs were also excluded. The treatment group received HM monotherapy or a combination of HM and conventional oral iron, whereas the control group primarily received oral iron only, sometimes in combination with vitamin supplements, folic acid, or dietary guidance to encourage consumption of iron-rich foods. When implementing dietary intervention, it was applied equally to both groups. Iron was administered orally only with no restrictions on the type of formulation, including powders, granules, syrups, solutions, capsules, or tablets.

#### 2.1.4 Outcome measures

The primary outcomes included the main IDA-related indicators, blood Hb, and SF levels. The secondary outcomes included the following:

1) Total effective rate (TER).
2) Incidence of adverse events (AEs) during treatment.
3) Average healing time.
4) Hematological markers: Total red blood cell (RBC) count and SI, MCV, MCH, and TIBC levels.

In case of TER indicator, the curative effectiveness was mostly classified into stages “cured” (N1), “markedly effective” (N2), “effective” (N3), and “ineffective” (N4). The TER was calculated as follows: {(N1 + N2 + N3)/total number of cases} × 100 (%). The criteria for clinical effectiveness differed slightly among studies. However, this was mainly based on the degree of improvement in blood markers, including Hb, after treatment.

### 2.2 Protocol registration

This systematic review and meta-analysis was based on the Preferred Reporting Items for Systematic Reviews and Meta-Analyses (PRISMA) guidelines (Moher et al., 2009). The research protocol has been registered on the PROSPERO platform (registration number CRD42022334670). It is available at: https://www.crd.york.ac.uk/prospero/display_record.php?ID=CRD42022334670.

### 2.3 Information sources and search strategy

Twelve databases were searched for clinical trials. The following databases were used:

1) Five English databases (PubMed, Embase, Elton B. Stephens Company [EBSCO], the Cochrane Central Register of Controlled Trials [CENTRAL], and the Allied and Complementary Medicine Database [AMED]).
2) Three Chinese databases (China National Knowledge Infrastructure [CNKI], Chinese Science and Technology Periodic Database [VIP], and Wangfang Database).
3) Three Korean databases (Oriental Medicine Advanced Searching Integrated System [OASIS], Korean studies Information Service System [KISS], and Korean Medical Database).
4) One Japanese database (Citation Information by National Institute of Informatics [CiNII]).

The search was conducted in 28 May 2023. The search strategy used a combination of medical subject headings and keywords for IDA. The strategy which was used in PubMed was as follows: {anemia, iron deficiency OR iron deficiency OR IDA OR hypochromic anemia OR [(ferrous OR ferric OR folic) AND anemia]} AND (child* OR pediatrics OR infant OR adolescent OR minors OR neonate OR newborn OR baby) AND (plants, medicinal OR drugs, Chinese herbal OR medicine, Chinese traditional OR medicine, kampo OR medicine, Korean traditional OR herbal medicine OR traditional oriental medicine OR herb* OR decoction* OR botanic*) AND (randomized controlled trial OR controlled clinical trial OR randomized OR drug therapy OR randomly OR trial OR groups) NOT (animals NOT humans). The entire search strategies are presented in Supplementary Table S1.

### 2.4 Study selection and data extraction

**FIGURE 1:**
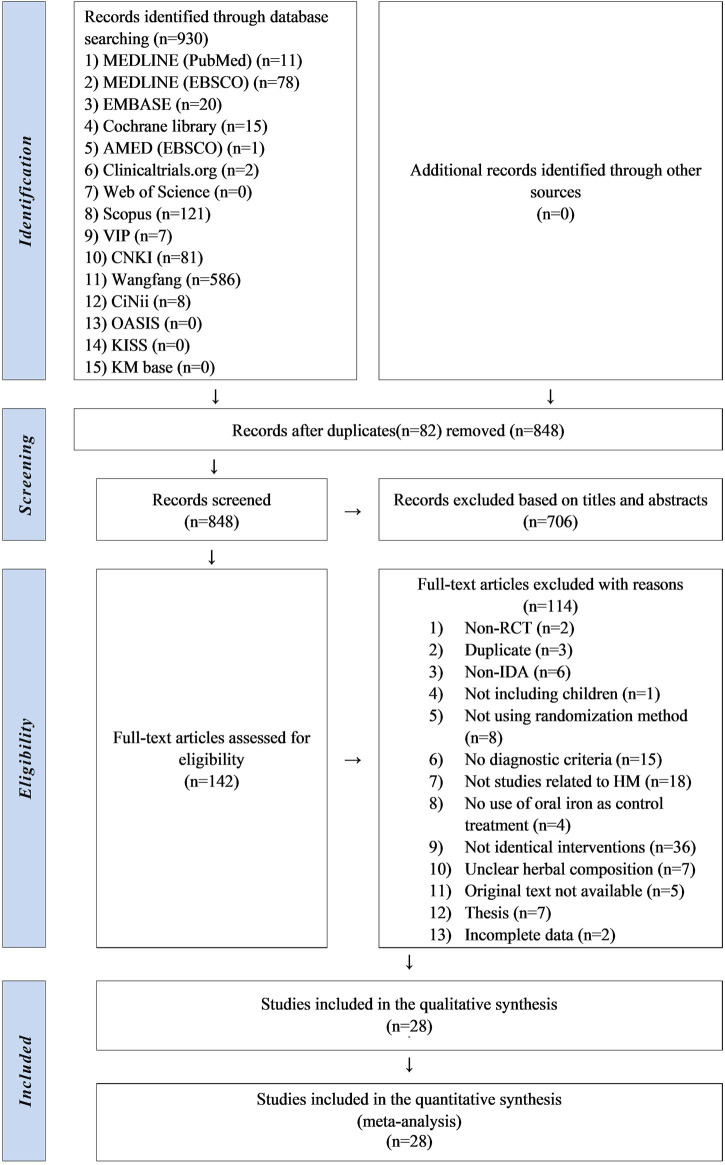
PRISMA flowchart of the literature selection process.

Two researchers independently selected and extracted the studies. Studies retrieved from each database were imported into the bibliographic information management program Endnote 20 (Clarivate Analytics, Philadelphia, PA, United States). After removing duplicates, titles and abstracts of the studies were screened. After eliminating irrelevant studies, a full-text review was conducted to identify studies that met the eligibility criteria. The excluded studies were categorized according to the reasons for exclusion. And the data was extracted according to the study characteristics (first author’s name, publication year, and country), participant characteristics (sample size, mean age or range, sex, and period of disease), intervention characteristics (type of intervention, prescription name, composition, dosage, treatment duration, frequency, and formulation), and clinical outcomes. Detailed data were recorded on a Microsoft Excel 2019 spreadsheet and are presented in Supplementary Tables S2, S3. All processes were cross-checked. If there were any concerns, we discussed with the corresponding author whether to include it. A diagram of the study selection process is presented in the PRISMA flowchart (Figure 1).

### 2.5 Risk of bias assessment

Two researchers independently assessed the risk of bias using the Cochrane Risk of Bias Assessment tool (Sterne et al., 2019). The domains of assessment were as follows: randomization process, deviations from intended interventions, missing outcome data, measurement of outcomes, selection of the reported result, and overall risk of bias. Each domain was evaluated as “low,” “some concern,” or “high” according to the criteria. In case of disagreement, a final decision was made after consultation with the corresponding author.

### 2.6 Statistical analysis

Review Manager 5.4.1 (Cochrane, London, United Kingdom) and meta packages (Lortie and Filazzola, 2020) in R studio (version 4.3.0) were used to conduct the meta-analysis. For dichotomous data synthesis, the risk ratio (RR) with a 95% confidence interval (CI) was adopted. For continuous data synthesis, the mean difference (MD) or standardized mean difference (SMD) with a 95% CI was adopted. Heterogeneity was evaluated using Higgins I^2^ statistics and *p*-values. If I^2^ ≥ 50% and *p* < 0.10 were found, a random-effect model was chosen considering a significant heterogeneity. A fixed-effects model was used for cases of I^2^ < 50% and *p* > 0.10. When significant heterogeneity was observed, subgroup analysis and meta-analysis of variance (meta-ANOVA) were performed according to the following moderator variables:

1) Type of intervention (HM vs. oral iron, HM plus oral iron vs. oral iron).
2) Duration of treatment (duration ≤30 days, 30 days < duration ≤60 days, 60 days < duration ≤90 days).

For outcomes that included more than 10 studies, publication bias was visually evaluated using a funnel plot. The asymmetry of the funnel plot was confirmed using Egger’s regression test (Egger et al., 1997), and the total effect size was re-evaluated after adjusting for asymmetry using the trim-and-fill method. Additionally, a sensitivity analysis was performed by excluding one study at a time to assess its impact on the effect size and to determine whether the overall effect size was still stable.

### 2.7 Quality of evidence assessment

The quality of evidence for the primary and secondary outcomes was evaluated using the Grading of Recommendations Assessment, Development, and Evaluation (GRADE) approach (Guyatt et al., 2008b) (http://gradepro.org). The quality of evidence was based on the following domains: risk of bias, inconsistency, indirectness, imprecision, and publication bias (Guyatt et al., 2008b). GRADE is categorized into four levels: very low, low, moderate, and high (Guyatt et al., 2008a). In the domain of inconsistency, if I^2^ was more than 75% and the effect sizes were in the same direction, the domain was evaluated as “serious,” whereas if I^2^ was more than 75% and the effect sizes were in different directions, the area was evaluated as “very serious.” In the domain of imprecision, if the sample size for a dichotomous variable did not meet the optimal information size, if the sample size for a continuous variable was less than 300, or if the 95% CIs of individual studies overlapped too broadly, the domain was rated as “serious,” and the level of evidence was downgraded.

## 3 Results

### 3.1 Study selection

In this review, 807 studies were initially identified through the database search. After excluding 80 duplicate studies, 727 studies were screened based on their titles and abstracts. A total of 585 studies were excluded and the full texts of the remaining 142 were reviewed. Of these, two were non-RCTs, three were duplicates, six did not concern IDA, one did not involve children, eight did not implement randomization allocation, 15 did not present diagnostic criteria, 18 were not related to HM, and four did not use oral iron therapy as a control treatment. In 36 studies, the interventions except for HM, oral iron, and vitamins were not identical between the groups. Seven studies did not report HM composition, five were not available in the original text, seven were theses, and two had incomplete data. Consequently, 28 studies (She, 2001; Li et al., 2003; Sun, 2004; Xie and Gao, 2006; Qin, 2007; Shao, 2010; Li and Luo, 2011; Sun, 2011; Zheng and Leng, 2011; Zhou, 2011; Xiao and Deng, 2012; Zhang, 2012; Tian, 2013; Lu, 2014; Feng, 2015; Gu, 2015; Li, 2015; Tan, 2015; Chen and Wang, 2017; Ni and Zhou, 2017; Wu, 2017; Chen, 2019; Deng, 2020; Hao et al., 2020; Li, 2021; Zhang and Xu, 2021; Chen et al., 2023; Qian et al., 2023) were finally selected (Figure 1).

### 3.2 Study characteristics

Of the 28 selected studies, 18 were retrieved from the Wangfang database and 10 from the CNKI. All studies were conducted in China and published between 2001 and 2023. A total of 3,044 children diagnosed with IDA were recruited, with 1,633 and 1,411 children assigned to the treatment and control groups, respectively.

Five studies (She, 2001; Sun, 2004; Shao, 2010; Sun, 2011; Zhou, 2011) compared the efficacy of HM monotherapy with that of oral iron, whereas 23 studies (Li et al., 2003; Xie and Gao, 2006; Qin, 2007; Li and Luo, 2011; Zheng and Leng, 2011; Xiao and Deng, 2012; Zhang, 2012; Tian, 2013; Lu, 2014; Feng, 2015; Gu, 2015; Li, 2015; Tan, 2015; Chen and Wang, 2017; Ni and Zhou, 2017; Wu, 2017; Chen, 2019; Deng, 2020; Hao et al., 2020; Li, 2021; Zhang and Xu, 2021; Chen et al., 2023; Qian et al., 2023) compared the efficacy of a combination of HM and oral iron with that of oral iron. The treatment duration ranged from 4 weeks to 3 months. When comparing the baseline characteristics of the patients before the intervention, no significant differences were found between the two groups in any of the studies (*p* > 0.05). The detailed characteristics of the included studies are presented in Supplementary Tables S2, S3.

Among the botanical prescriptions used to treat IDA in children and adolescents, Xingpi Yanger granules (Li et al., 2003; Li and Luo, 2011; Xiao and Deng, 2012; Tian, 2013; Lu, 2014) were the most commonly reported prescription in five studies. Qixue granules (Shao, 2010; Sun, 2011; Zhou, 2011; Li, 2015) were reported in four studies, whereas modified Shenling Baizhu powder (She, 2001; Ni and Zhou, 2017; Chen, 2019) and Danggui Buxue decoction (Sun, 2004; Qin, 2007; Chen and Wang, 2017) were reported in three studies each. Jianpi Yiqi Shengxue decoction (Wu, 2017; Chen et al., 2023) was reported in two studies. The remaining studies reported different prescriptions.

Among the botanical drugs included in the prescription, *Astragali Radix*, the dried root of *Astragalus mongholicus Bunge [Fabaceae]* was most frequently reported (21 studies), followed by *Atractylodis Rhizoma Alba,* the dried rhizome of *Atractylodes macrocephala Koidz. [Asteraceae]* in 15, *Angelicae Sinensis Radix*, the dried root of *Angelica sinensis (Oliv.) Diels [Apiaceae]* in 14, *Citri Unshius Pericarpium*, the dried pericarp of the ripe fruit of *Citrus reticulata Blanco [Rutaceae]* in 13, and *Poria Sclerotium*, a medicinal fungus of *Wolfiporia extensa (Peck) Ginns [Polyporaceae]* in 12 (Table 1).

**TABLE 1:** Top five East Asian botanical drugs used to treat iron deficiency anemia in children.

Number of studies (%)	Accepted name [Family; Latin name]
21 (75)	*Astragalus mongholicus Bunge [Fabaceae; Astragali Radix]*
15 (54)	*Atractylodes macrocephala Koidz. [Asteraceae; Atractylodis Rhizoma Alba]*
14 (50)	*Angelica sinensis (Oliv.) Diels [Apiaceae; Angelicae Sinensis Radix]*
13 (46)	*Citrus reticulata Blanco [Rutaceae; Citri Unshius Pericarpium]*
12 (43)	*Wolfiporia extensa (Peck) Ginns [Polyporaceae; Poria Sclerotium]*

### 3.3 Risk of bias assessment

**FIGURE 2:**
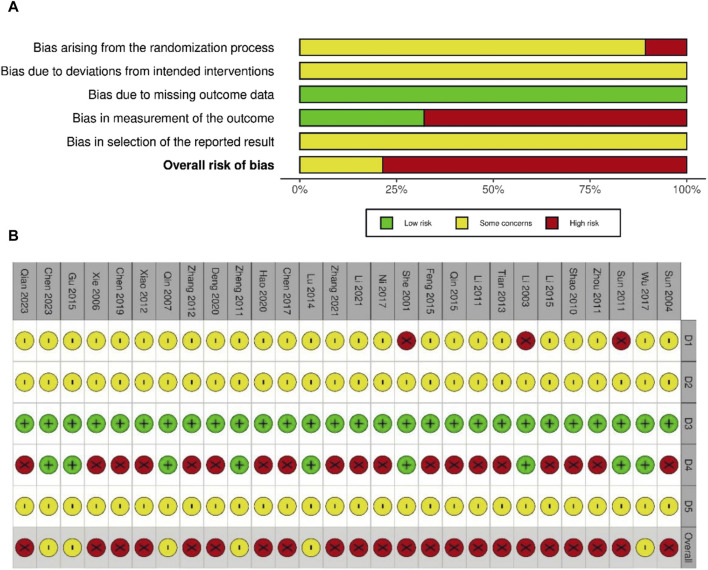
Risk of bias graph **(A)** and summary of risk of bias **(B)**. D1: bias arising from the randomization process; D2: bias due to deviations from intended interventions; D3: bias due to missing outcome data; D4: bias in measurement of the outcome; D5: bias in selection of the reported results. ⊕ (Green), low risk; ⊗ (red), high risk; Θ (yellow), some concerns.

The risk of bias was assessed for all 28 studies. No study showed a low level of risk, whereas six (Qin, 2007; Zheng and Leng, 2011; Lu, 2014; Gu, 2015; Wu, 2017; Chen et al., 2023) showed some concerns regarding the level of risk, and 22 showed a high level of risk. All studies mentioned randomization when assigning patients to treatment and control groups. However, 18 studies did not describe the randomization method in detail, except for 10 studies (Tian, 2013; Gu, 2015; Li, 2015; Tan, 2015; Deng, 2020; Hao et al., 2020; Li, 2021; Zhang and Xu, 2021; Chen et al., 2023; Qian et al., 2023), which used the random number table method. None of the studies mentioned information on blinding, and three (She, 2001; Li et al., 2003; Sun, 2011) were evaluated as high risk in the domain of randomization owing to a large imbalance in the number of patients between groups. Moreover, 19 studies (Sun, 2004; Xie and Gao, 2006; Shao, 2010; Li and Luo, 2011; Zhou, 2011; Xiao and Deng, 2012; Zhang, 2012; Tian, 2013; Feng, 2015; Li, 2015; Tan, 2015; Chen and Wang, 2017; Ni and Zhou, 2017; Chen, 2019; Deng, 2020; Hao et al., 2020; Li, 2021; Zhang and Xu, 2021; Qian et al., 2023) that reported subjective AE symptoms as an outcome were assessed as having a high risk of bias because they did not conceal the intervention and the knowledge of the intervention could have influenced the assessment. The risks of bias are summarized in Figures 2A, B.

### 3.4 Meta-analysis results

#### 3.4.1 Primary outcomes

##### 3.4.1.1 Hemoglobin

**FIGURE 3:**
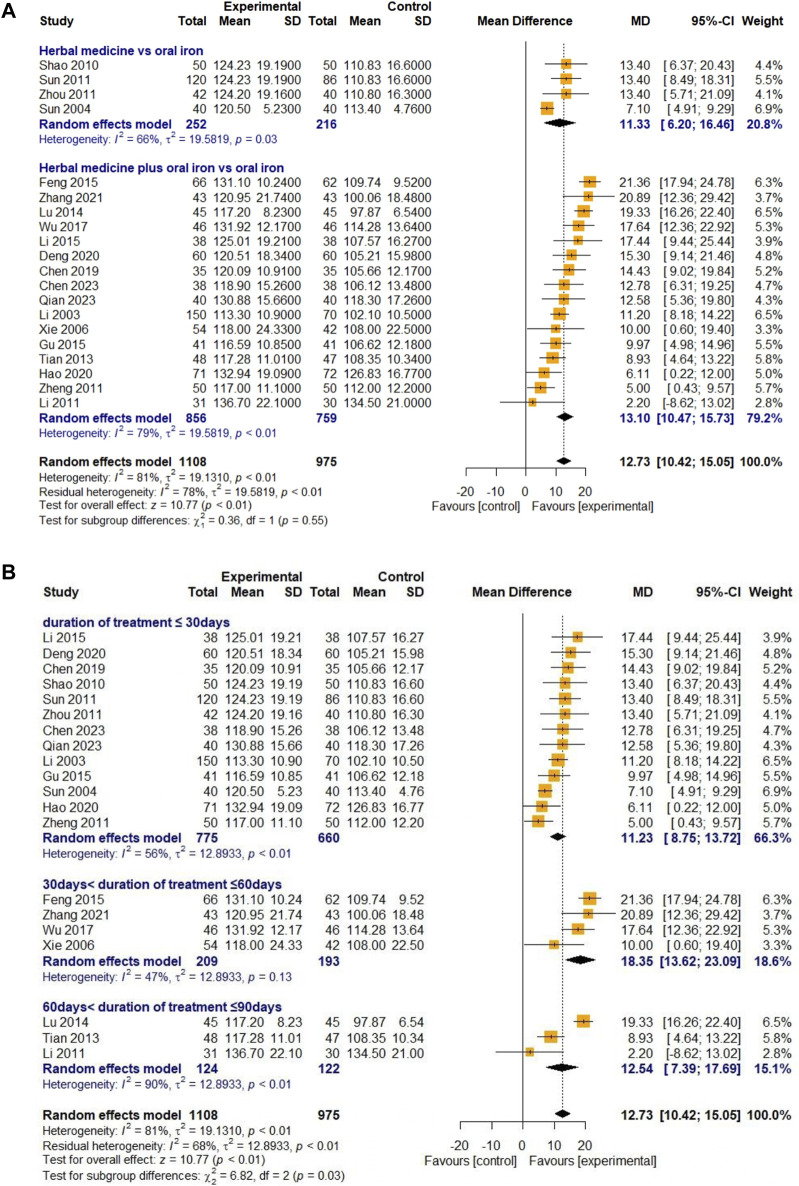
Forest plot comparing the effect of HM with that of oral iron on Hb (meta-ANOVA) by intervention type **(A)** and treatment duration **(B)**.

Twenty studies were selected to evaluate blood Hb levels. Four studies reported on HM monotherapy and 16 studies on combination therapy with oral iron. A total of 1,108 and 975 children with IDA were recruited into treatment and control groups, respectively. For high heterogeneity (I ² = 81%, *p* < 0.01), a random-effects model was used for evaluation. The overall effect of HM on Hb levels was significantly better than that in the control group [MD 12.73, 95% CI (10.42; 15.05), Z = 10.77, *p* < 0.01]. In subgroup analysis, the efficacy of HM in the combination group with oral iron [MD 13.10, 95% CI (10.47; 15.73)] was greater than that in the HM monotherapy group [MD 11.33, 95% CI (6.20; 16.46)], but the difference between groups was not significant (χ ² = 0.36, df = 1, *p* = 0.55) (Figure 3A). An additional subgroup analysis and meta-ANOVA for the duration of treatment revealed significant differences in the increase in Hb levels between the subgroups compared to those in the control group (χ ² = 6.82, df = 2, *p* = 0.03). The increase in Hb levels was the greatest in the group with 30–60 days of treatment compared to that in the control group [MD 18.35, 95% CI (13.62; 23.09)] (Figure 3B). Egger’s regression test showed no publication bias (t = 0.51, df = 18, *p* = 0.6174) (Figure 10A). The sensitivity analysis also showed that the overall results were stable after eliminating the studies individually (Figure 11A).

##### 3.4.1.2 Serum ferritin

**FIGURE 4:**
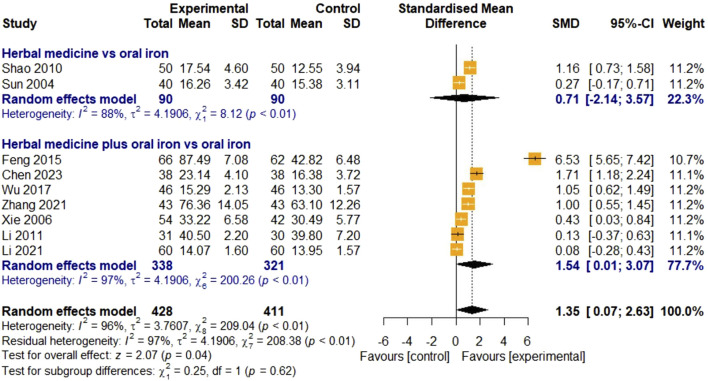
Forest plot comparing the effect of HM with that of oral iron on SF (meta-ANOVA).

The SF levels were evaluated in nine studies. Two studies were on HM monotherapy, and seven were on HM in combination with oral iron. In total, 428 and 411 children with IDA were enrolled in the treatment and control groups, respectively. For high heterogeneity (I ² = 96%, *p* < 0.01), a random-effects model was used. The overall effect of HM on SF was statistically significant compared with that in the control group [SMD 1.35, 95% CI (0.07; 2.63)], Z = 2.07, *p* = 0.04). Subgroup analysis showed that the effect of HM monotherapy was comparable to that of oral iron [SMD 0.71, 95% CI (-2.14; 3.57)]. However, the effect of the combination of HM and oral iron was significantly better than that of the control group [SMD 1.54, 95% CI (0.07; 2.63)] (Figure 4). To explain the high heterogeneity, we performed additional subgroup analyses and meta-ANOVA by treatment duration. However, no significant subgroup difference was identified in treatment duration (χ ² = 1.35, df = 2, *p* = 0.51). Publication bias analysis was not performed because the number of included studies was less than 10. Furthermore, in the sensitivity analysis, the results of the meta-analysis remained stable after removing studies individually (Figure 11B).

#### 3.4.2 Secondary outcomes

##### 3.4.2.1 Total effective rate

**FIGURE 5:**
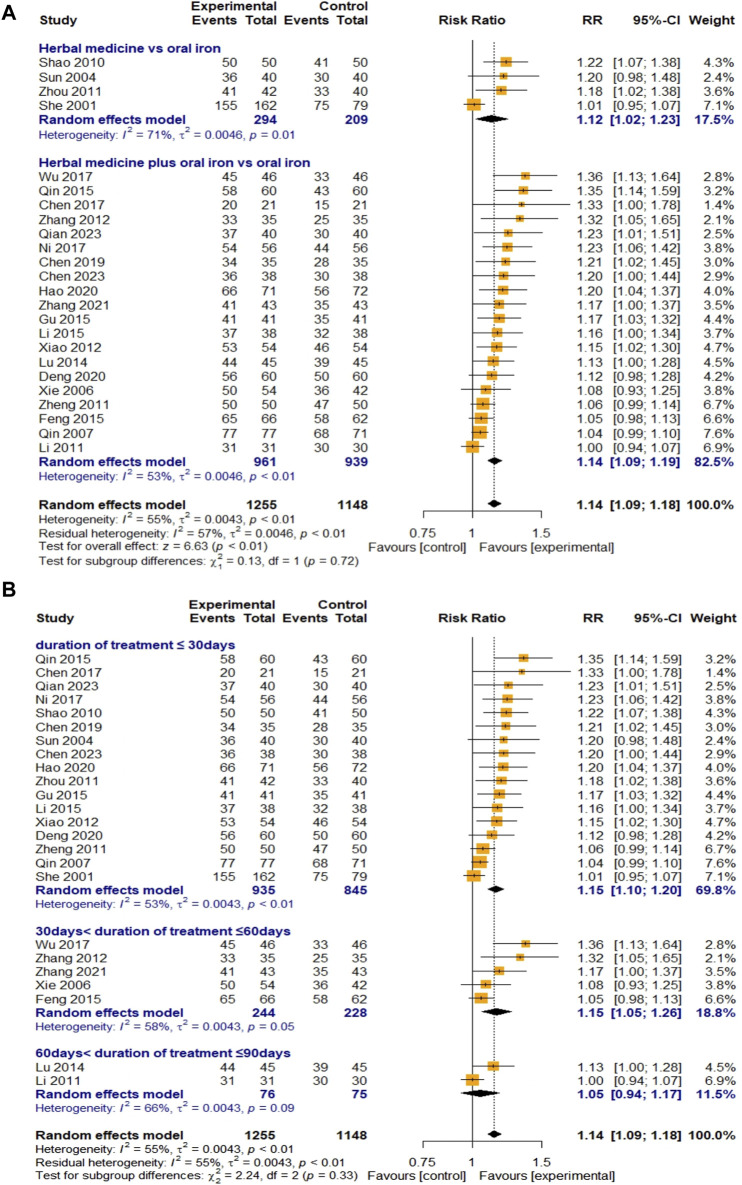
Forest plot comparing the effect of HM with that of oral iron on TER (meta-ANOVA) by intervention type **(A)** and treatment duration **(B)**.

A total of 24 studies evaluated the efficacy of HM based on the TER. Four studies were on HM monotherapy, and 20 were on combination therapy with oral iron. A total of 1,255 and 1,148 children with IDA were recruited for the treatment and control groups, respectively. The overall efficacy of HM on TER was statistically significant compared to that of oral iron (RR 1.14, 95% CI [1.09; 1.18], Z = 6.63, *p* < 0.01). Owing to the substantial heterogeneity (I ² = 55%, *p* < 0.01), a random-effects model was used for the meta-analysis. The meta-ANOVA results showed that there was no significant difference in the efficacy of HM monotherapy and that of combination therapy with oral iron (χ ² = 0.13, df = 1, *p* = 0.72) (Figure 5A). Further analysis revealed that the high heterogeneity in the HM monotherapy group (I ² = 71%, *p* = 0.01) can be attributed to the difference in herbal prescription by She (2001) (She, 2001), as the other three studies on monotherapy used similar types of HM derived from one regimen. Additional subgroup analyses and meta-ANOVA on the treatment duration showed no significant differences among subgroups (χ ² = 2.24, df = 2, *p* = 0.33). However, it showed that for less than 60 days, HM was superior to oral iron in TER (RR 1.15), whereas for a treatment duration of 60–90 days, the effect of HM was similar to that of oral iron treatment [RR 1.05, 95% CI (0.93; 1.18)] (Figure 5B). Egger’s regression test showed that publication bias might have existed (t = 9.47, df = 22, *p* < 0.0001). However, after the trim-and-fill method by adding 11 studies that were assumed to be missing, the result remained robust [RR 1.07, 95% CI (1.03; 1.12), *p* = 0.001] (Figure 10B). The sensitivity analysis also showed that the overall results remained stable after eliminating the studies individually (Figure 11C).

##### 3.4.2.2 Red blood cell count

**FIGURE 6:**
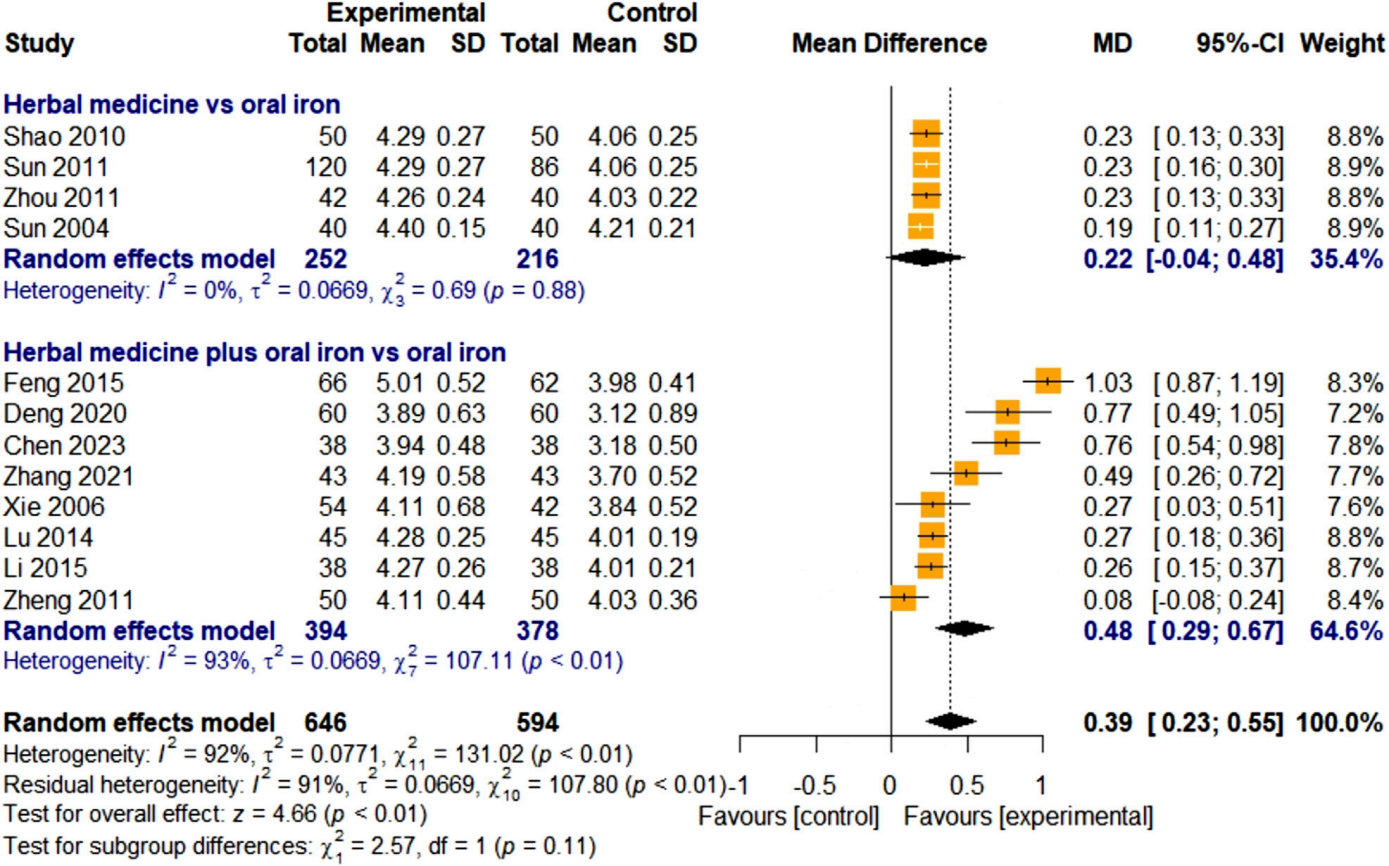
Forest plot comparing the effect of HM with that of oral iron on RBCs (meta-ANOVA).

Twelve studies assessed the efficacy of HM using RBC counts. Four studies focused on HM monotherapy and eight studies focused on combination therapy with HM and oral iron. In total, 646 and 594 children with IDA were assigned to the treatment and control groups, respectively. The overall effect of HM on RBC levels was significant, compared with that of the control group [MD 0.39, 95% CI (0.23; 0.55), Z = 4.66, *p* < 0.01]. Because of the overall high heterogeneity (I ² = 92%, *p* < 0.01), the random-effects model was selected and subgroup analyses were conducted. Meta-ANOVA showed no significant subgroup difference between HM monotherapy and combination therapy with oral iron (χ ² = 2.57, df = 1, *p* = 0.11). In HM monotherapy, the effect was slightly better than that of oral iron [MD 0.22, 95% CI (-0.04; 0.48)], but this was not statistically significant (*p* = 0.88), whereas HM combination therapy was significantly more effective than the control [MD 0.48, 95% CI (0.29; 0.67), *p* < 0.01] (Figure 6). Due to the high heterogeneity in the HM and oral iron combination group (I^2^ = 93%, *p* < 0.01), subgroup analysis by treatment duration was conducted for the combination group. However, the result showed no significant difference between subgroups (χ ² = 2.94, df = 2, *p* = 0.23). Egger’s regression test showed that there might have been publication bias (*p* = 0.0458). However, the results proved to be robust when the trim-and-fill method was applied including three studies that were thought to be missing [MD 0.24, 95% CI (0.03; 0.45), *p* = 0.0249] (Figure 10C). Moreover, the sensitivity analysis showed that all results were stable after removing each study individually (Figure 11D).

##### 3.4.2.3 Serum iron

**FIGURE 7:**
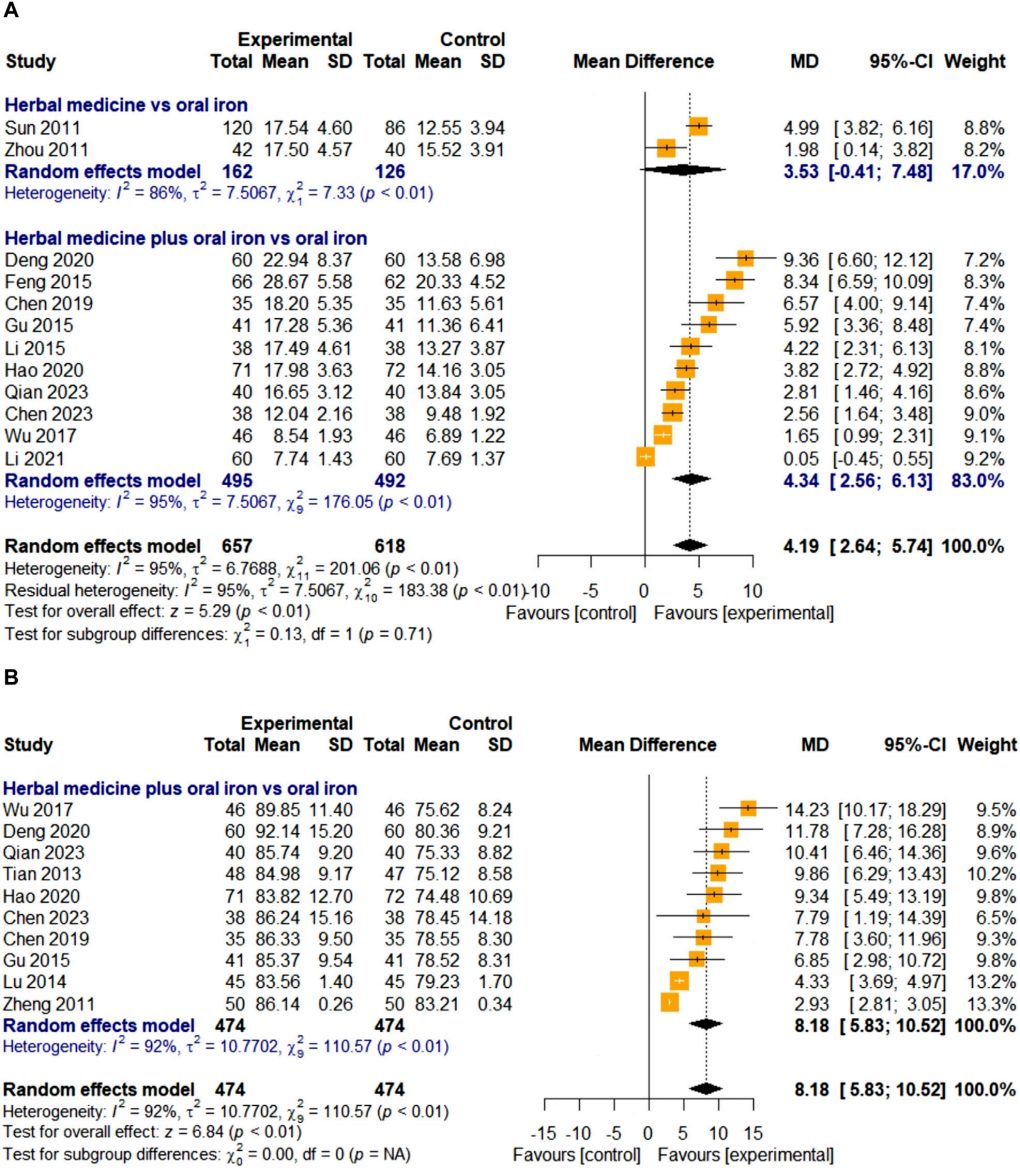
Forest plot comparing the effect of HM with that of oral iron on SI **(A)**, and MCV **(B)**.

The efficacy of HM on SI was evaluated in 12 studies. Two studies were treated with HM alone, and 10 studies were treated with a combination therapy of HM and oral iron. In total, 657 and 618 children with IDA were assigned to the treatment and control groups, respectively. The overall effect of HM on the SI levels was significant compared to that of oral iron [MD 4.19, 95% CI (2.64; 5.74)]. For high heterogeneity (I ² = 95%, *p* < 0.01), a random-effects model was selected, and subgroup analyses were performed according to the three moderator variables. Meta-ANOVA showed that there was no significant difference in the effects between groups receiving HM alone and those receiving HM in combination with oral iron (χ ² = 0.13, df = 1, *p* = 0.71). However, HM combined with oral iron increased SI levels more effectively than HM alone [MD 4.34, 95% CI (2.56; 6.13) in the combination group; MD 3.53, 95% CI (-0.41; 7.48) in the HM alone group] (Figure 7A). Additional subgroup analysis by treatment duration did not reveal any significant difference between groups (χ ² = 0.13, df = 1, *p* = 0.72); however, the effect of HM was greater in the group treated for 30–60 days [MD 4.85, 95% CI (0.95; 8.75)] than that in the groups treated for less than 30 days [MD 4.07, 95% CI (2.29; 5.85)]. The linear regression test for funnel plot asymmetry indicated possible publication bias (*p* = 0.0005). Therefore, we recalculated the effect size using the trim-and-fill method by adding six studies that were purportedly missing; the result was not robust [MD 1.6189, 95% CI (-0.51; 3.75), *p* = 0.1361] (Figure 10D). Sensitivity analyses were also performed using the leave-one-out method, and all results were stable (Figure 11E).

##### 3.4.2.4 Mean corpuscular volume

Ten studies evaluated the efficacy of HM on MCV. All studies used HM plus oral iron as the treatment intervention, and the overall effect of HM was significantly better than those of the treatments in the control group [MD 8.18, 95% CI (5.83; 10.52), Z = 6.84, *p* < 0.01] (Figure 7B). A total of 474 children with IDA were enrolled in the treatment and control groups. Because of the high heterogeneity (I ² = 92%, *p* < 0.01), the random-effects model was chosen and meta-ANOVAs were conducted as subgroup analyses. Further subgroup analysis on the treatment duration showed that HM was significantly more effective than oral iron in all subgroups. However, meta-ANOVA did not reveal any significant subgroup differences (χ ² = 3.21, df = 2, *p* = 0.20). Egger’s regression test indicated the possibility of publication bias (*p* < 0.0001). After performing a trim-and-fill method by adding six studies, the result was robust [MD 3.61, 95% CI (0.11; 7.12), *p* = 0.0431] (Figure 10E). Similarly, the sensitivity test results were stable (Figure 11F).

##### 3.4.2.5 Mean corpuscular hemoglobin

**FIGURE 8:**
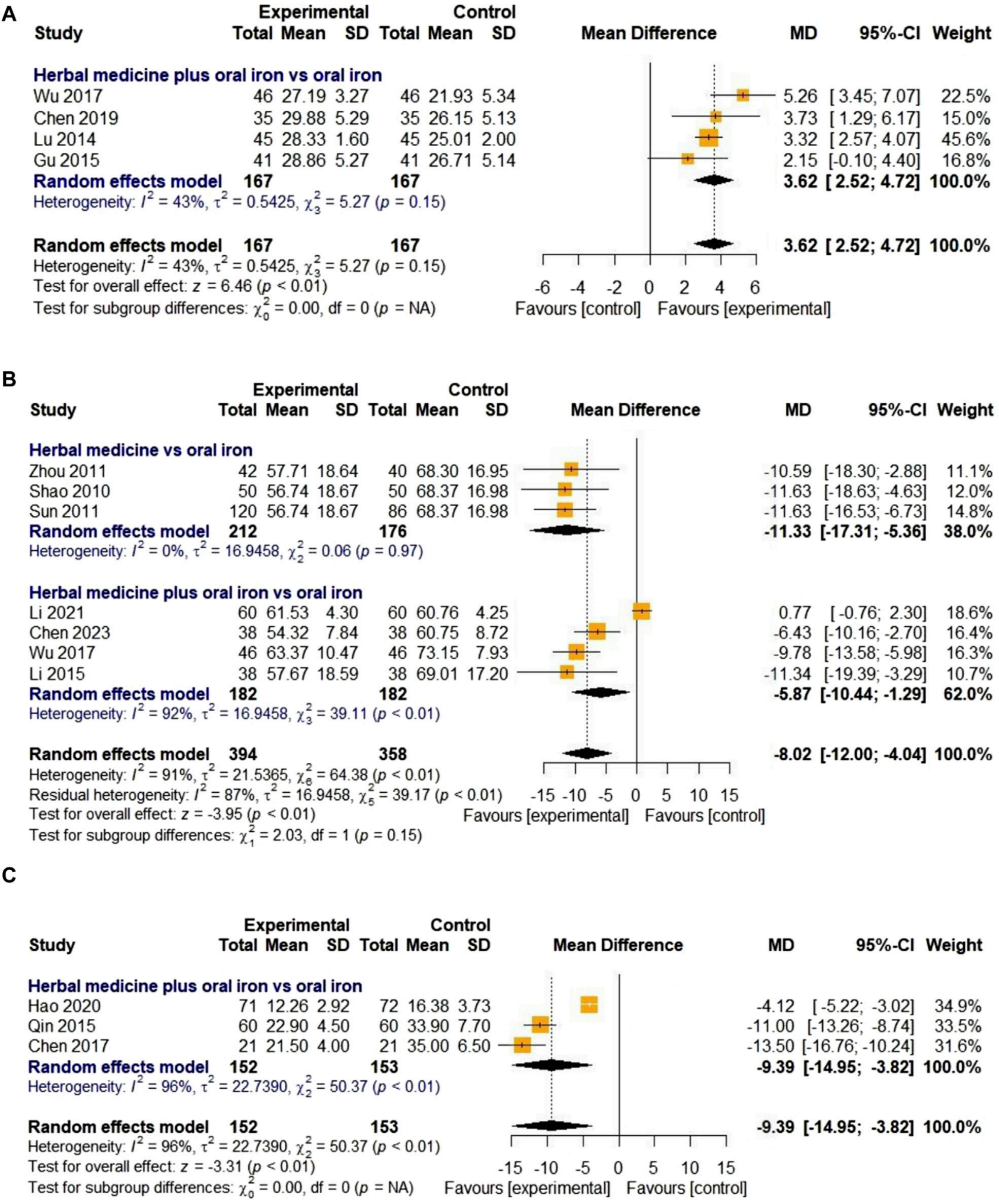
Forest plot comparing the effect of HM with that of oral iron on MCH **(A)**, TIBC **(B)**, and average healing time (days) **(C)**.

Four studies evaluated the effect of HM on MCH levels. They were all about combination therapies with oral iron, not HM monotherapy. In total, 167 children with IDA were enrolled in the treatment and control groups. The overall effect of HM in combination with oral iron on MCH levels was significantly better than that of the treatments in the control group [MD 3.62, 95% CI (2.52; 4.72), Z = 6.46, *p* < 0.01]. As the heterogeneity was lower than 50% (I ² = 43%, *p* = 0.15), a fixed-effects model was used, and subgroup analyses were not performed (Figure 8A). Egger’s regression test was not conducted because the number of included studies was less than 10. The results of the sensitivity analysis, which were meta-analyzed by removing studies one by one, were also stable (Figure 11G).

##### 3.4.2.6 Total iron binding capacity

Seven studies were included to evaluate the effect of HM on TIBC levels. Three and four studies on HM monotherapy and HM combined therapy with oral iron, respectively, were considered. The treatment and control groups included 394 and 358 children with IDA, respectively. The overall effect in the treatment group was statistically significant compared to that in the control group [MD -8.02, 95% CI (-12.00; −4.04), Z = −3.95, *p* < 0.01]. Due to high heterogeneity (I ² = 91%, *p* < 0.01), a random-effects model was selected and subgroup analyses of the three moderator variables were performed. In the first subgroup meta-analysis, the HM monotherapy reduced TIBC levels more effectively [MD -11.33. 95% CI (-17.31; −5.36)] than the effect of combination therapy with oral iron [MD -5.87, 95% CI (-10.44; −1.29)]. However, meta-ANOVA showed no significant differences between subgroups (χ ² = 2.03, df = 1, *p* = 0.15) (Figure 8B). Further subgroup analysis on treatment duration also revealed no significant difference between subgroups (χ ² = 0.43, df = 1, *p* = 0.51). We did not perform Egger’s regression test for publication bias because the number of included studies was less than 10, and the sensitivity analyses performed by removing studies individually were stable (Figure 11H).

##### 3.4.2.7 Average healing time

Only three studies evaluated the clinical efficacy of HM in relation to the average healing time. In one study (Hao et al., 2020), the disappearance time of clinical symptoms was described, whereas the other two studies only described the healing time (days) and did not provide a detailed definition. All three studies compared HM plus oral iron to oral iron alone, with the treatment lasting for 30 days. Due to the high heterogeneity (I ² = 96%, *p* < 0.01), a random-effects model was applied. After a 30-day treatment, the average healing time in the treatment group was significantly shorter than that in the control group, and the difference was statistically significant [MD -9.39, 95% CI (-14.95; −3.82), Z = −3.31, *p* < 0.01] (Figure 8C). Because the number of included studies was less than 10, a test for publication bias was not performed. Sensitivity analysis showed that all results were stable when meta-analyses were conducted by removing the studies one by one (Figure 11J).

##### 3.4.2.8 Incidence of adverse events

Nineteen studies evaluated the effectiveness of HM in reducing AE incidence. Most AEs reported in the treatment and control groups were gastrointestinal symptoms, including nausea, vomiting, epigastric discomfort, and constipation.

**FIGURE 9:**
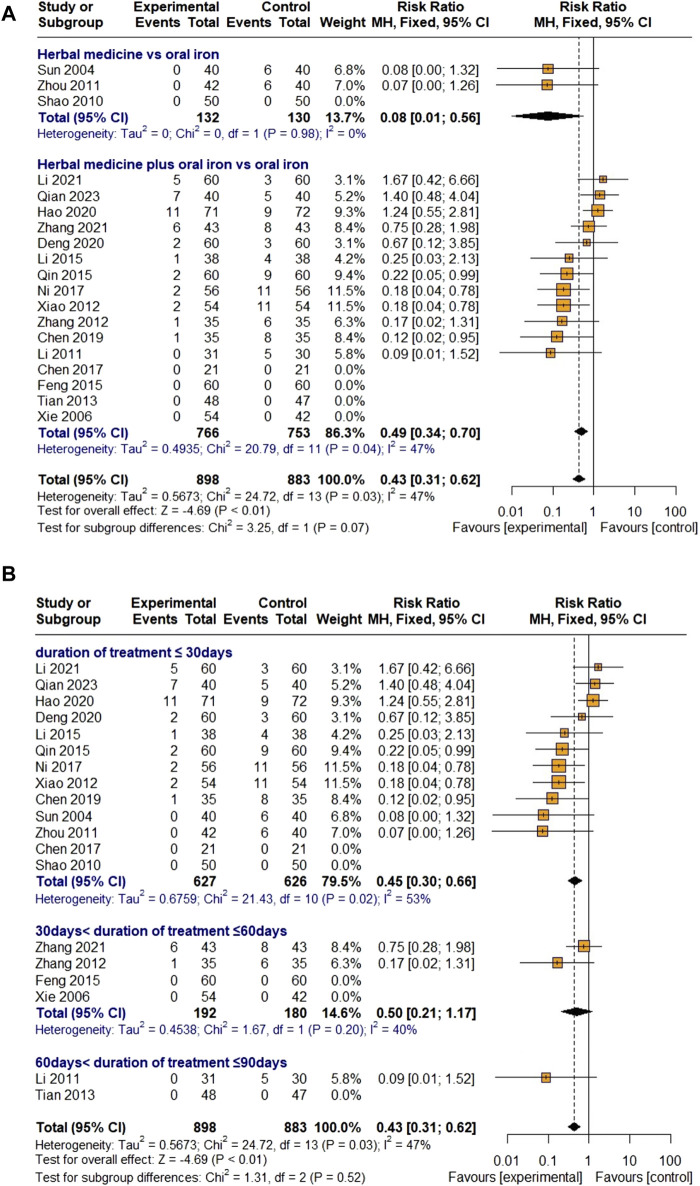
Forest plot comparing the effect of HM with that of oral iron on AEs (meta-ANOVA) by intervention type **(A)** and treatment duration **(B)**.

There were three studies on HM monotherapy and 16 studies on combination therapy with oral iron, with a total of 898 and 883 children with IDA assigned to the treatment and control groups, respectively. As heterogeneity was lower than 50% (I ² = 47%, *p* = 0.03), a fixed-effects model was applied, and the overall effect of HM on AEs was found to be statistically significant compared with that of the control group [RR 0.43, 95% CI (0.31; 0.62), Z = −2.96, *p* < 0.01]. Although meta-ANOVA revealed no significant difference between HM monotherapy and HM and oral iron combined therapy (χ ² = 3.25, df = 1, *p* = 0.07), the AEs were less common in the HM monotherapy group [RR 0.08, 95% CI (0.01; 0.56)] compared to the combination group with oral iron [RR 0.49, 95% CI (0.34; 0.70)] (Figure 9A).

**FIGURE 10:**
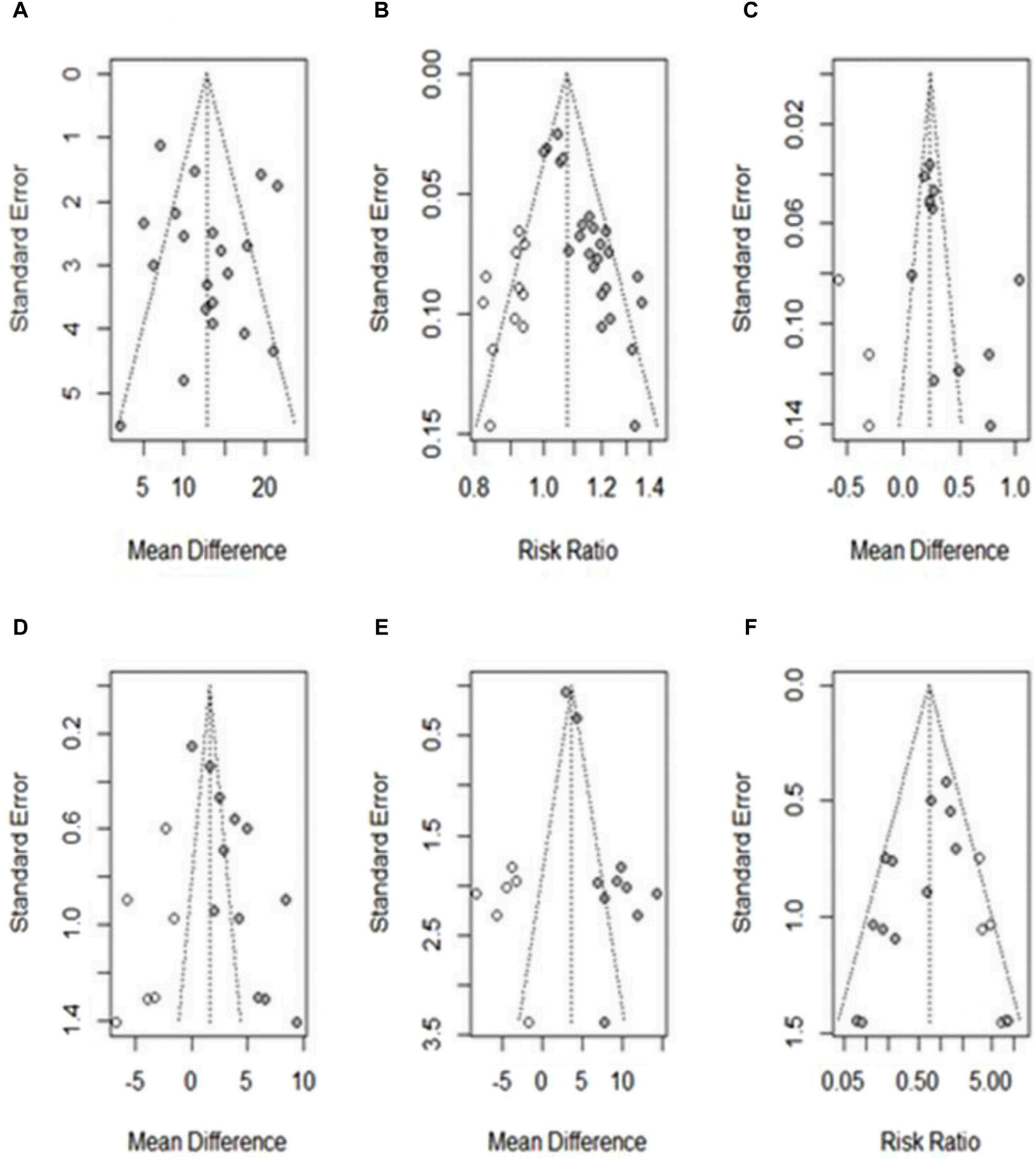
Funnel plot showing publication bias after applying the trim-and-fill method. **(A)** Hb; **(B)** TER; **(C)** RBCs; **(D)** SI; **(E)** MCV; **(F)** AE. ○: studies believed to be missing; ●: studies included in the outcome.

**FIGURE 11:**
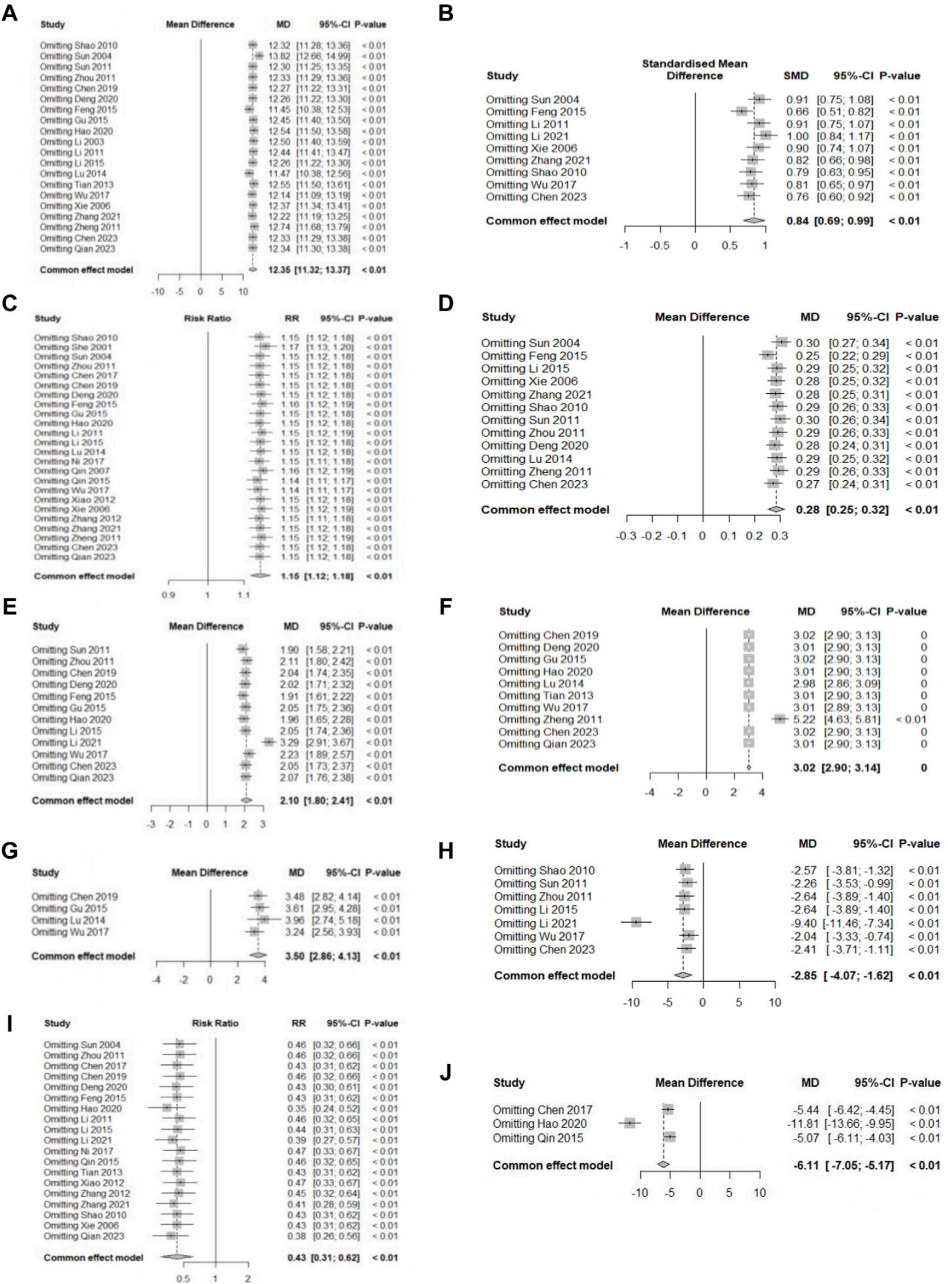
Sensitivity analysis plot. **(A)** Hb; **(B)** SF; **(C)** TER; **(D)** RBC; **(E)** SI; **(F)** MCV; **(G)** MCH; **(H)** TIBC; **(I)** AE; **(J)** Average healing time.

Further subgroup analysis by treatment duration showed that HM had a significant effect on reducing AEs in the groups treated for less than 30 days [RR 0.45, 95% CI (0.30; 0.66)] and reduced the incidence of AEs in the groups treated for 30–60 days [RR 0.50, 95% CI (0.21; 1.17)], but the difference was not significant (Figure 9B). Because Egger’s regression test suggested a publication bias (*p* = 0.0007), a trim-and-fill method was performed by removing five studies that did not report AEs and adding six studies that were thought to be missing. However, the result was not robust [RR 0.71, 95% CI (0.39; 1.30), Z = −1.11, *p* = 0.2691] (Figure 10F). Sensitivity analyses were performed by serially eliminating studies, and the results were stable (Figure 11I).

#### 3.4.3 Other outcomes

This study assessed additional outcome indicators, such as mean corpuscular hemoglobin concentration (MCHC) or recurrence rate, as well as those analyzed above. However, except for MCHC and recurrence rate, the additional outcomes were not meta-analyzed separately because of the small number of included studies. The most frequently reported MCHC levels were found in nine studies, with a statistically significant increase in MCHC level when HM plus oral iron was compared with oral iron alone [SMD 1.04, 95% CI (0.89; 1.19), Z = 13.69, *p* < 0.01]. The second most frequently reported additional outcome was the recurrence rate, which was reported in three studies, including one study of HM monotherapy and two studies on combination therapy with oral iron. The meta-analysis showed a statistically significant reduction in the recurrence rate after HM treatment [RR 0.20, 95% CI (0.08; 0.53), Z = 3.24, *p* = 0.001]. Additional outcomes included weight, TCM syndrome score, TS levels, hematocrit, total renal blood flow, and reticulocyte levels. The results are summarized in Supplementary Table S1.

#### 3.4.4 East Asian botanical prescriptions including *Astragali Radix*

**FIGURE 12:**
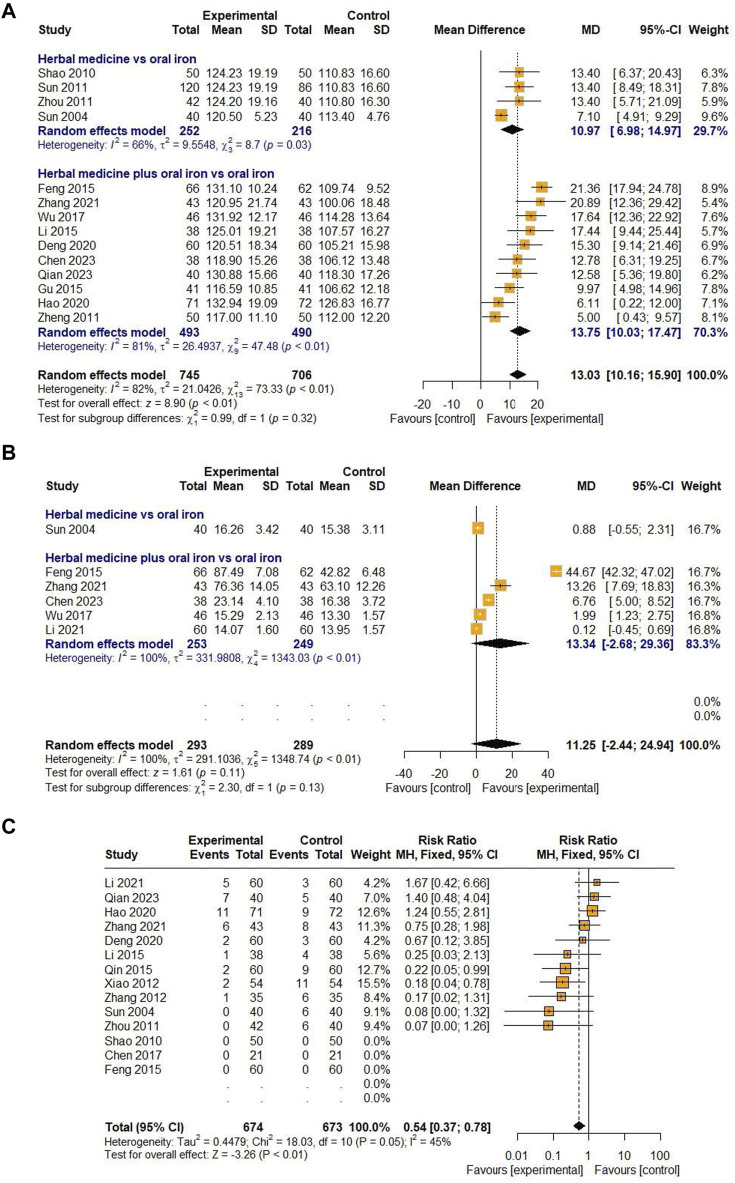
Forest plot comparing the effect of HM including *Astragali Radix* on Hb **(A)**, SF **(B)**, and AE **(C)**.

*Astragali Radix* was the most frequently reported botanical drug (21 studies, 75%) among the prescriptions included in the study. Therefore, we performed an additional meta-analysis to investigate the efficacy and safety of East Asian HM containing *Astragali Radix*. The indicators meta-analyzed were Hb and SF levels as the primary outcome and the incidence of AEs as the secondary outcome. As a result, we found out significant effects of HMs containing *Astragali Radix* on increasing Hb levels [MD 13.03, 95% CI (10.16; 15.90), Z = 8.90, *p* < 0.01] and reducing AEs [RR 0.54, 95% CI (0.37; 0.78), Z = −3.26, *p* < 0.01] in children with IDA (Figure 12).

### 3.5 Publication bias

For outcomes with more than 10 studies, publication bias was assessed using funnel plots and Egger regression tests (Egger et al., 1997). Among the 10 outcomes, there was no publication bias in the studies on Hb levels. Egger’s regression test showed the potential for publication bias in studies related to TER, RBC, and MCV levels; however, the overall effect sizes remained robust after adjustment using the trim-and-fill method. The risk of publication bias was also found in studies on SI levels and AE incidence; however, the overall effect was not robust after re-evaluation using the trim-and-fill method. Other outcomes were not assessed for publication bias because fewer than 10 studies were included. The funnel plots for each outcome are shown in Figures 10A–F.

### 3.6 Sensitivity analysis

Sensitivity analyses were performed for all primary and secondary outcomes, and all results remained stable (*p* < 0.01) when the effect sizes were recalculated after excluding one study at a time. The detailed results are presented in Figures 11A–J.

### 3.7 Quality of evidence

The quality of evidence was evaluated using GRADEpro. In this systematic review, the GRADE evaluation was divided into two groups: HM versus oral iron and HM plus oral iron versus oral iron. In the HM monotherapy group, the outcomes for Hb, TER, RBC, and incidence of AEs were rated as “moderate.” They were downgraded in the risk of bias domain owing to selection, performance, and detection bias. SF and SI were graded “very low” due to risk in domains of inconsistency and imprecision. In the combination group of HM and oral iron, several outcomes (TER, SF, RBC, MCH) were rated as “low,” whereas the other outcomes were rated as “very low.” The results and details of the level of evidence for GRADE are presented in Supplementary Table S4.

## 4 Discussion

### 4.1 Main findings and clinical implications

This study conducted a systematic review and meta-analysis to evaluate the efficacy and safety of East Asian HM for the treatment of IDA in children. A total of 28 RCTs were meta-analyzed, including five studies comparing the efficacy of HM alone with that of oral iron alone and 23 studies comparing the efficacy of a combination of HM and oral iron with that of oral iron alone.

As the primary outcomes, Hb level, which determines anemia, and SF level, which is the most accurate indicator of iron status (Cappellini et al., 2020) were selected, and the secondary outcomes included TER, blood markers related to IDA, incidence of AEs, and average healing time from IDA to compare the differences between groups after treatment. The treatment and control groups were not significantly different before treatment. However, after treatment with HM, the results showed that the overall effect of the treatment group was higher than that of the control group in terms of all outcome measures (*p* < 0.05). In addition, the main hematologic markers, TER, and mean recovery time were all significant in the treatment group, indicating the superior efficacy of East Asian HM compared with that of oral iron in the treatment of IDA in children. Regarding the AEs related to the safety assessment, HM significantly reduced the incidence of gastrointestinal AEs, compared with oral iron (RR 0.43). Therefore, HM may be considered more effective and safer than oral iron for treating IDA.

Among the 28 included studies, five reported the efficacy of HM monotherapy, and all used oral iron as a control treatment. The oral iron used was ferrous fumarate, ferrous sulfate, iron dextran, and ferric glycerophosphate. In some studies, vitamins were taken with iron or dietary guidance was given to eat iron-rich foods equally in both groups. The treatment duration in the HM monotherapy studies was either 4 weeks or 30 days, and all outcomes (Hb, TER, TIBC, and RBC) were significantly improved in the HM treatment group compared to the oral iron group, except for SF and SI levels, which were reported in two studies. The incidence of AEs was also significantly reduced in the HM group compared to the control group, supporting the efficacy and safety of HM as monotherapy.

Twenty-three studies reported the efficacy of the combination therapy with HM and oral iron. The treatment group received both HM and oral iron, whereas the control group received only the same oral iron as the treatment group. The oral iron used was ferrous fumarate, ferrous sulfate, ferrous succinate, ferrous gluconate, ferrous lactate, ferrous fumarate, iron dextran, and iron protein succinylate. Similarly, some studies have added the same vitamins or dietary guidance to both groups. The HM and oral iron combination treatment group showed significant improvements in all outcome measures compared to those observed in the control group, suggesting the effectiveness of HM as a combination therapy. Furthermore, the combination therapy group reported significantly fewer AEs than those reported by the control group. The symptoms reported as AEs in both groups were mostly gastrointestinal symptoms, such as vomiting, abdominal pain, diarrhea, and constipation. As gastrointestinal AEs are one of the main factors that reduce adherence to oral iron treatment in children with IDA (Andersen et al., 2023), it is important to evaluate whether HM is significantly safer than oral iron. Although the meta-ANOVA revealed no significant difference between the HM monotherapy and combination groups (*p* = 0.07), significantly fewer AEs were reported in both the HM alone and HM plus oral iron treatment groups compared to those reported in the oral iron groups. This showed that HM not only significantly reduced gastrointestinal side effects, but also had a significantly better safety than that of oral iron.

In this study, meta-analyses were performed on all orally administered HMs compared with oral iron rather than dividing HMs into specific prescriptions. Therefore, heterogeneity may exist depending on the type and formulation of HM. However, to minimize heterogeneity, meta-ANOVA was performed as subgroup analysis on two moderator variables (type of intervention and treatment duration). Although the individual meta-ANOVAs yielded inconsistent results across all outcome measures, the evidence for the efficacy and safety of HMs for IDA treatment in children was obtained via detailed analyses on the moderator variables.

First, a meta-analysis was conducted by dividing treatment duration into less than 30, 30–60, and 60–90 days to determine whether HM treatment was superior to oral iron in a relatively short period of time. The results showed that when HM was administered for less than 2 months, most outcomes improved significantly (Hb, TER, RBC, SI, MCV, TIBC, and average healing time from IDA) compared to the improvements observed when oral iron alone was administered. In addition, the results also showed that HM treatment was more effective in the groups treated for 30–60 days than in the groups treated for less than 30 days on many outcome measures (TER, RBC, SI), including hemoglobin, the primary outcome. This suggests that the efficacy of oral iron, which requires relatively long-term treatment, catches up with that of HM after 2 months, or that the efficacy of HM treatment of 60–90 days may be underestimated, as only three studies with a treatment duration of 60–90 days were included in this review. In conclusion, HM improved blood markers, increased TER, and shortened the average healing time in a relatively short period compared with oral iron, supporting its advantage in terms of treatment duration.

Furthermore, this study analyzed the prescriptions of HM used in children with IDA and ordered the prescription names and botanical drugs by frequency. Among the prescriptions, the most frequently used prescriptions were Xingpi Yanger Granules (5 studies, 17.86%), Qixue granules (4 studies, 14.29%), and modified Shenling Baizhu powder and modified Danggui Buxue decoction (3 studies, 10.71% in each study). According to one study (Sun, 2011), Qixue granules are a prescription derived from the Danggui Buxue decoction, and HM similar to Danggui Buxue decoction, which contains *Astragali Radix* and *Angelicae Sinensis Radix* as the main botanical drugs, was frequently used to treat IDA in this review. The most frequently used botanical drug was *Astragali Radix* (21 studies, 75%), followed by *Atractylodis Rhizoma Alba*, *Angelicae Sinensis Radix, Citri Unshius Pericarpium,* and *Poria Sclerotium* (Table 1). *Astragali Radix* is an important qi-tonifying medicinal botanical drug that strengthens the flow of qi in the lungs and spleen (Chen and Wang, 2017) and promotes erythrocyte glycolysis (Xu et al., 1987). *Angelicae Sinensis Radix* has blood-enhancing effects (Chen and Wang, 2017), such as promoting angiogenesis (Meng et al., 2006). Combining these two botanical drugs can improve the symptoms of blood deficiency and qi weakness. According to a rat study, a combination group treated with *Astragali Radix* and *Angelicae Sinensis Radix* showed significantly enhanced levels of blood markers and Hb concentrations compared to those observed in the control group (Chang et al., 2009).

In TCM, anemia is categorized as “deficiency syndrome,” especially blood deficiency (Ding et al., 2019), with clinical manifestations including pale and sallow complexion, loss of appetite, fatigue, lack of energy, inability to concentrate, and pale tongue with white and thin fur (She, 2001; Sun, 2011; Zhou, 2011; Zhang, 2012; Feng, 2015; Tan, 2015; Chen and Wang, 2017; Ni and Zhou, 2017; Wu, 2017; Chen et al., 2023; Qian et al., 2023). Clinical syndrome differentiation includes spleen-kidney yang deficiency, dual deficiency of the heart-spleen, and spleen-stomach weakness (Chen and Wang, 2017). Since these are also broad patterns of qi deficiency, it is understandable that *Astragali Radix* was frequently used to treat IDA in this research. Furthermore, the additional meta-analysis revealed the significant efficacy of HM containing *Astragali Radix* in increasing the Hb levels and reducing the incidence of AEs. Therefore, based on these findings, we hope that further studies, such as RCTs, could be conducted in clinical practice to establish a statistical basis for the use of HM containing *Astragali Radix* in children and adolescents with IDA.

This review analyzed 28 RCTs on botanical drugs to determine the efficacy and safety of HM compared with oral iron, revealing the potential of HM treatment as an alternative to oral iron treatment for IDA in children. And this study is important because it analyzed the effect of all HMs for the treatment of IDA in children according to the three moderator variables, and it analyzed high-frequency types of botanical prescriptions and individual botanical drugs used in the treatment of IDA.

### 4.2 Limitations of the study

The research only included databases from Western countries, such as the United States and Europe, and East Asian countries, such as Korea, China, and Japan, and did not include databases from Southeast Asian countries, such as India or Bangladesh. In addition, the studies finally included in this review were all conducted in China; therefore, its findings may be difficult to generalize. Since allocation concealment and blinding were not explicitly mentioned in any of the RCTs, it is possible that the efficacy of HM in the Chinese pediatric population could be overestimated, given that subjects in China are more likely to be familiar with East Asian traditional medicine such as HM compared to Western countries. Furthermore, the fact that most studies were considered to have a high risk of bias also contributed to the downgrading of the level of evidence. And many RCTs were small-studies with limited sample sizes, which may imply a risk of imprecision in individual study effects. In addition, because this was a comprehensive meta-analysis of the efficacy of all botanical drugs, rather than specific prescriptions, in improving IDA-related blood markers and reducing adverse events, there was often substantial heterogeneity despite subgroup analyses. Therefore, based on the findings of this study, further studies need to include individual meta-analyses of frequently used prescriptions with similar botanical composition and mechanism of action to reduce bias in the results. And in the future, it is necessary to conduct high-quality and large-scale studies that adhere to allocation concealment and blinding to reduce the risk of bias and minimize heterogeneity. Obtaining high-quality evidence from large-scale RCTs would strengthen the supporting evidence for the use of botanical drugs to treat IDA in children and increase confidence in the reproducibility of the results.

## 5 Conclusion

This meta-analysis identified evidence for the efficacy and safety of HM in children with IDA. Compared to conventional oral iron therapy, HM improved IDA-related blood markers and TER, with fewer AEs and shorter average healing times. However, well-designed and large-scale clinical trials are required to strengthen the supporting evidence for the efficacy and safety of HM for the treatment of IDA in children.

## Data Availability

The original contributions presented in the study are included in the article/Supplementary Material, further inquiries can be directed to the corresponding author.
