# Supplementary material for: Safety and efficacy of East Asian herbal medicine for iron deficiency anemia in children and adolescents: a systematic review and meta-analysis
Source: Front Pharmacol. 2024 Apr 8;15:1339486. doi: 10.3389/fphar.2024.1339486 (PMC11036862; doi:10.3389/fphar.2024.1339486)
Supplement: Supplementary file 1 [file Table1.DOCX]

Supplementary Material

# Supplementary Table 1. Search strategies for each database

- CNKI

|  | Searches | Results |
| --- | --- | --- |
| #1 | (SU='贫血'+‘贫血症’+'缺铁性贫血'+‘小细胞’+‘低色素性’+‘铁质缺乏’) AND (SU='小儿'+'儿童'+'儿'+'小孩'+'孩子') AND (SU='中医药'+'中医'+'中西医结合'+'中药'+'汤'+'丸'+'散'+'饮'+'颗粒'+'胶囊'+'自拟') AND (SU='随机'+'对照'+'随意'+'试验'+'安慰') | 81 |

- Pubmed

|  | Searches | Results |
| --- | --- | --- |
| #1 | "Anemia, iron deficiency"[MH] OR "iron deficiency an?emia"[TIAB] OR "iron-deficiency an?emia"[TIAB] OR “iron deficien*”[TIAB] OR “iron-deficien*”[TIAB] OR (“iron”[TIAB] AND “deficien*”[TIAB] AND “an?emia”[TIAB]) OR ((“microcytic“[TIAB] OR ”hypochromic”[TIAB]) AND “anemia”[TIAB]) OR　“IDA”[MH] OR (("iron"[TIAB] OR “ferritin”[TIAB] OR “ferrous”[TIAB] OR “ferric*”[TIAB] OR “fe”[TIAB] OR “folic*”[TIAB] OR “folate*“[TIAB] OR “folvite*“[TIAB] OR “folacin*“[TIAB] OR “pteroylglutamic*“[TIAB]) AND “anemia“[TIAB] | 24,347 |
| #2 | Child[MH] OR Pediatrics[MH] OR Infant[MH] OR Adolescent[MH] OR Minors[MH] OR child*[TIAB] OR pediatric*[TIAB] OR infant[TIAB] OR neonate[TIAB] OR newborn[TIAB] OR adolescent[TIAB] OR baby[TIAB] | 4,403,377 |
| #3 | “Plants, Medicinal”[MH] OR “Drugs, Chinese Herbal”[MH] OR “Medicine, Chinese Traditional”[MH] OR “Medicine, Kampo”[MH] OR “Medicine, Korean Traditional”[MH] OR “Herbal Medicine”[MH] OR “traditional Korean medicine”[TIAB] OR “traditional Chinese medicine”[TIAB] OR “traditional oriental medicine”[TIAB] OR “Kampo medicine”[TIAB] OR herb*[TIAB] OR decoction*[TIAB] OR botanic*[TIAB] | 243,649 |
| #4 | “Randomized controlled trial”[PT] OR “controlled clinical trial”[PT] OR randomized[TIAB] OR placebo[TIAB] OR “drug therapy”[SH] OR randomly[TIAB] OR trial[TIAB] OR groups[TIAB] | 5,437,843 |
| #5 | Animals[MH] NOT humans[MH] | 5,009,560 |
| #6 | #1 AND #2 AND #3 AND #4 NOT #5 | 11 |

- EMBASE via Elsevier

|  | Searches | Results |
| --- | --- | --- |
| #1 | ‘Iron deficiency anemia’/exp OR ‘iron-deficiency anemia’/exp OR 'iron deficien*'/exp OR ‘iron-deficien*’/exp OR (‘iron’/exp AND ‘deficien*’/exp AND 'an$emia*'/exp) OR ((‘microcytic anemia’/exp OR ‘hypochromic’/exp) AND ’anemia’/exp) OR ‘iron deficiency an$emia*’/exp OR ‘anemia iron deficiency therapy’/exp OR ‘IDA’/exp OR ((‘iron’/exp OR ‘ferritin’/exp OR ‘ferrous’/exp OR ‘ferric*’/exp OR ‘fe’/exp OR ‘folic$’/exp OR ‘folate$’/exp OR ‘folvite$’/exp OR ‘folacin$’/exp OR ‘pteroylglutamic$’/exp) AND ‘anemia’/exp) | 114,300 |
| #2 | Child/exp OR pediatrics/exp OR infant/exp OR adolescent/exp OR ‘minor (person)’/exp OR child*:ab,ti OR pediatric*:ab,ti OR infant:ab,ti OR neonate:ab,ti OR newborn:ab,ti OR adolescent:ab,ti OR baby:ab,ti | 4,798,825 |
| #3 | ‘Medicinal plant’/exp OR ‘medicinal plant’:ab,ti OR ‘herbaceous agent’/exp OR ‘herbaceous agent’:ab,ti OR ‘chinese medicine’/exp OR ‘chinese medicine’:ab,ti OR ‘kampo medicine’/exp OR ‘kampo medicine’:ab,ti OR ‘kampo medicine (drug)’/exp OR ‘kampo medicine (drug)’:ab,ti OR ‘korean medicine’/exp OR ‘korean medicine’:ab,ti OR ‘herbal medicine’/exp OR ‘herbal medicine’:ab,ti OR ‘oriental medicine’/exp OR ‘oriental medicine’:ab,ti OR herb/exp OR herb*:ab,ti OR decoction*:ab,ti OR botanic*:ab,ti | 489,457 |
| #4 | ‘Crossover procedure’:de OR ‘double-blind procedure’:de OR ‘randomized controlled trial’:de OR ‘single-blind procedure’:de OR (random* OR factorial* OR crossover* OR cross NEXT/1 over* OR placebo* OR doubl* NEAR/1 blind* OR singl* NEAR/1 blind* OR assign* OR allocat* OR volunteer*):de,ab,ti | 2,938,524 |
| #5 | #1 AND #2 AND #3 AND #4 | 36 |
| #6 | #5 AND 'randomized controlled trial'/de | 20 |

- CENTRAL (Cochrane)

|  | Searches | Results |
| --- | --- | --- |
| #1 | MeSH descriptor: [Anemia, Iron‐Deficiency] explode all trees | 1,450 |
| #2 | MeSH descriptor Iron, this term only | 156 |
| #3 | MeSH descriptor Iron, Dietary, this term only | 65 |
| #4 | (((Iron* OR folic* OR ferritin OR ferrous* OR ferric* OR fe OR folic* OR folate* OR folvite* OR folacin*) AND anemia) OR iron deficien* OR iron-deficien* OR　(iron AND deficien* AND an?emia*)):ti,ab,kw | 6,942 |
| #5 | ((Microcytic anemia OR hypochromic) AND anemia):ti,ab,kw | 323 |
| #6 | #1 OR #2 OR #3 OR #4 OR #5 | 7,087 |
| #7 | MeSH descriptor: [Child] explode all trees | 61,040 |
| #8 | MeSH descriptor: [Pediatrics] explode all trees | 724 |
| #9 | MeSH descriptor: [Infant] explode all trees | 34,717 |
| #10 | MeSH descriptor: [Adolescent] explode all trees | 110,030 |
| #11 | MeSH descriptor: [Minors] explode all trees | 11 |
| #12 | (Child* OR pediatric* OR infant OR neonate OR newborn OR adolescent OR baby OR babies OR teen* OR adolescen* OR preteen* OR youth* OR young person* OR young people):ti,ab,kw | 313,531 |
| #13 | #7 OR #8 OR #9 OR #10 OR #11 OR #12 | 313,539 |
| #14 | MeSH descriptor: [Plants, Medicinal] explode all trees | 951 |
| #15 | MeSH descriptor: [Drugs, Chinese Herbal] explode all trees | 3,777 |
| #16 | MeSH descriptor: [Medicine, Chinese Traditional] explode all trees | 1,260 |
| #17 | MeSH descriptor: [Medicine, Kampo] explode all trees | 48 |
| #18 | MeSH descriptor: [Medicine, Korean Traditional] explode all trees | 33 |
| #19 | MeSH descriptor: [Herbal Medicine] explode all trees | 66 |
| #20 | (“Traditional Korean medicine” OR “traditional Chinese medicine” OR “traditional oriental medicine” OR “Kampo medicine” OR herb* OR decoction* OR botanic*):ti,ab,kw | 19,910 |
| #21 | #14 OR #15 OR #16 OR #17 OR #18 OR #19 OR #20 | 21,084 |
| #22 | (#6 AND #13 AND #21) in Trials | 15 |

- AMED via EBSCO

|  | Searches | Results |
| --- | --- | --- |
| #1 | Anemia, iron deficiency[SU] OR iron deficiency an#emia[SU] OR　iron-deficiency an#emia[SU] OR iron deficien*[SU] OR iron-deficien*[SU] OR　IDA[SU] OR ((microcytic[SU] OR hypochromic[SU] OR iron deficien*[SU] OR iron[SU] OR ferritin[TX] OR ferrous[TX] OR ferric*[TX] OR fe[TX] OR folic*[TX] OR folate[TX] OR folvite[TX] OR folacin*[TX]) AND an#emia[SU]) | 125 |
| #2 | Child[SU] OR Pediatrics[SU] OR Infant[SU] OR Adolescent[SU] OR Minors[SU] OR child*[TX] OR pediatric*[TX] OR infant[TX] OR neonate[TX] OR newborn[TX] OR adolescent[TX] OR baby[TX] | 32,793 |
| #3 | Plants, Medicinal[SU] OR Drugs, Chinese Herbal[SU] OR Medicine, Chinese Traditional[SU] OR Medicine, Kampo[SU] OR Medicine, Korean Traditional[SU] OR Herbal Medicine[SU] OR traditional Korean medicine[TX] OR traditional Chinese medicine[TX] OR traditional oriental medicine[TX] OR Kampo medicine[TX] OR herb*[TX] OR decoction*[TX] OR botanic*[TX] | 36,668 |
| #4 | Randomized controlled trial OR controlled clinical trial OR randomized crossover procedure OR double-blind procedure OR randomized controlled trial OR single-blind procedure OR random* OR factorial* OR crossover* OR placebo* OR double blind* OR assign* OR allocat* OR volunteer* | 32,080 |
| #5 | #1 AND #2 AND #3 AND #4 | 1 |

- MEDLINE via EBSCO

|  | Searches | Results |
| --- | --- | --- |
| S8 | S4 AND S5 AND S6 AND S7 | 78 |
| S7 | Randomized controlled trial OR controlled clinical trial OR randomized crossover procedure OR double-blind procedure OR randomized controlled trial OR single-blind procedure OR random* OR factorial* OR crossover* OR placebo* OR double blind* OR assign* OR allocat* OR volunteer* | 2,124,772 |
| S6 | SU (Plants, Medicinal OR Drugs, Chinese Herbal OR Medicine, Chinese Traditional OR Medicine, Kampo OR Medicine, Korean Traditional OR Herbal Medicine) OR TX (traditional Korean medicine OR traditional Chinese medicine OR traditional oriental medicine OR Kampo medicine OR herb* OR decoction* OR botanic*) | 662,807 |
| S5 | SU (Child OR Pediatrics OR Infant OR Adolescent OR Minors) OR TX (child* OR pediatric* OR infant OR neonate OR newborn OR adolescent OR baby) | 6,305,150 |
| S4 | S1 OR S3 | 55,639 |
| S3 | S2 AND SU an#emia | 36,068 |
| S2 | SU (microcytic OR hypochromic OR iron deficien* OR iron) OR TX (ferritin OR ferrous OR ferric* OR fe OR folic* OR folate OR folvite OR folacin*) | 779,034 |
| S1 | SU anemia, iron deficiency OR iron deficiency an#emia OR　iron-deficiency an#emia OR iron deficien* OR iron-deficien* OR IDA OR (SU (iron AND deficien* AND an#emia ) | 37,186 |

- OASIS

|  | Searches | Results |
| --- | --- | --- |
| #1 | (빈혈 OR 철결핍성 OR 철) (소아 OR 아동) (한 OR 약초 OR 본초 OR 탕 OR 환 OR 산) | 0 |

- KMbase

|  | Searches | Results |
| --- | --- | --- |
| #1 | ((([TITLE=빈혈] OR [TITLE=철결핍]) OR [TITLE=철분]) OR [TITLE=철]) | 1576 |
| #2 | ([ABSTRACT=소아] OR [ABSTRACT=아동]) | 8997 |
| #3 | ((((([ABSTRACT=한약] OR [ABSTRACT=약초]) OR [ABSTRACT=본초]) OR [ABSTRACT=탕]) | 735 |
| #4 | #1 AND #2 AND #3 | 0 |

- KISS

|  | Searches | Results |
| --- | --- | --- |
| #1 | Title =(빈혈\|철결핍\|철분\|철) AND Abstract=(소아\|아동) AND Abstract =(한약\|약초\|본초\|탕\|환\|산) | 0 |

- Wanfang

|  | Searches | Results |
| --- | --- | --- |
| #1 | TI=(贫血 OR 贫血症 OR 缺铁性贫血 OR 低色素性 OR 铁质缺乏) AND (TI=(小儿 OR 儿童 OR 儿 OR 小孩 OR 孩子) OR AB=(小儿 OR 儿童 OR 儿 OR 小孩 OR 孩子)) AND (TI=(中医药 OR 中医 OR 中西医结合 OR 中药 OR 汤 OR 丸 OR 散 OR 饮 OR 颗粒 OR 胶囊 OR 自拟) OR AB=(中医药 OR 中医 OR 中西医结合 OR 中药 OR 汤 OR 丸 OR 散 OR 饮 OR 颗粒 OR 胶囊 OR 自拟)) AND (TI=(随机 OR 对照 OR 随意 OR 试验 OR 安慰) OR AB=(随机 OR 对照 OR 随意 OR 试验 OR 安慰)) | 586 |

- VIP

|  | Searches | Results |
| --- | --- | --- |
| #1 | (M=(贫血 OR 贫血症 OR 缺铁性贫血 OR 铁质缺乏) AND M=(小儿 OR 儿童 OR 儿 OR 小孩 OR 孩子) AND M=(中医药 OR 中医 OR 中西医结合 OR 中药 OR 汤 OR 丸 OR 散 OR 饮 OR 颗粒 OR 胶囊 OR 自拟) AND M=(随机 OR 对照 OR 随意 OR 试验 OR 安慰)) | 7 |

- CiNII

|  | Searches | Results |
| --- | --- | --- |
| #1 | (贫血 OR 贫血症 OR 缺铁性贫血 OR 铁质缺乏) AND (小児 OR 幼子 OR 児子 OR 子 OR 児 OR 児童 OR 幼児 OR 乳兒 OR 子供 OR 思春期の OR 青春の) AND (漢方薬 OR ハーブ OR 散 OR 汤 OR 丸) AND (ランダム化比較試験 OR 対照臨床試験 OR ランダム OR 無作為 OR 対照 OR 試験 OR 偽薬) | 8 |

# Supplementary Table 2. Characteristics of the included studies

| **Study ID** | **Sample size (M:F);**  **Mean age or range (y)** | | **Interventions** | | **Treatment duration** | | **Outcome** | **Intergroup comparison** | **Adverse events (number)** | | | |
| --- | --- | --- | --- | --- | --- | --- | --- | --- | --- | --- | --- | --- |
|  | **T** | **C** | **T** | **C** |  |  |  |  | **T** | **C** | | |
| **East Asian herbal medicine versus oral iron** | | | | | | | | | | | | |
| She 2001 | 162(84:78)  ;4.79±1.23  (0.5-8y) | 79(41:38)  ;4.83±1.25 (0.5-9y) | 1. Modified Shenling Baizhu powder  (<6y: 1/4-1/3 pk, tid or qid, ≥6y: 1/3-1/2 pk, bid or tid) | 1. Ferrous fumarate  (20 mg/(kg·d), tid) | 4 w | 1. TER  2. Improvement of spleen and stomach weakness  3. Urinary D-xylose excretion rate  4. Urine amylase activity  5. Recurrence rate | | 1. NS  2. Anorexia, diarrhea: T>C+, pale and sallow complexion, fatigue, pale tongue and lips: NS  3. T>C+  4. T>C+  5. T<C* | NR | NR | |  |
| Sun 2004 | 40(23:17)  ;0.5-7y | 40(26:14)  ;0.6-7y | 1. Modified Danggui Buxue decoction (1pk=1000mL)  (<3y: 1/200 pk, ≥3y: 1/100 pk, tid)  2.Rountine feeding | 1. Ferrous sulfate (20 % iron content)  (0.02g/kg, tid)  2. Vitamin C  (0.1 g, tid)  3. Rountine feeding | 1 mo | 1. TER  2. RBC  3. Hb  4. SF  5. AE | | 1. T>C*  2. T>C+  3. T>C+  4. T>C*  5. NS | None | Gastrointestinal discomfort, nausea, vomiting, anorexia, abdominal pain and diarrhea (6) |  |  |
| Shao 2010 | 50 | 50 | 1. Qixue granules  (1 bag, tid) | 1. Oral iron dextran (500 mg/d, tid) | 30 d | 1. TER  2. Hb  3. RBC  4. SF  5. TIBC  6. AE | | 1. T>C+  2. T>C+  3. T>C+  4. T>C+  5. T<C+  6. Not estimable | None | None |  |  |
|  | 100(46:54)  ;4.18 (1-12y) | |  |  |  |  |  |  |  |  |  |  |
| Sun 2011 | 120 | 86 | 1. Qixue Granules  (1-2 bag/d, tid)  2. Dietary guidance (add food rich in iron) | 1. Multivital iron oral solution (containing ferric glycerophosphate 5 mg per 1 mL, vitamin C 100 mg, vitamin B2 1.5 mg, etc.)  2. Dietary guidance (to add food rich in iron) | 4 w | 1. RBC  2. Hb  3. SI  4. TIBC | | 1. T>C+  2. T>C+  3. T>C+  4. T<C+ | NR | NR |  |  |
|  | 206(136:70)  ;7mo-14y | |  |  |  |  |  |  |  |  |  |  |
| Zhou 2011 | 42 | 40 | 1. Qixue granules  (1 bag, tid, pc) | 1. Oral iron dextran dispersible tablets  (50 mg, tid) | 30 d | 1. RBC  2. Hb  3. SI  4. TIBC  5. TER  6. AE | | 1. T>C+  2. T>C+  3. T>C+  4. T<C+  5. T>C*  6. NS | None | Metallic taste, gastrointestinal irritation, nausea, vomiting, abdominal pain (6) |  |  |
|  | 82(53:29)  ;0.5-14y | |  |  |  |  |  |  |  |  |  |  |
| **East Asian herbal medicine plus oral iron versus oral iron** | | | | | | | | | | | | |
| Li 2003 | 150(82:68)  ;11.2 mo  (0.5-2y) | 70(38:32)  ;10.8 mo  (0.5-2y) | 1. Xingpi Yanger granules  (<1y: 2 g, 1-2y: 4 g, bid)  2. (C) | 1. Ferrous fumarate granules  (<1y: 0.1 g, 1-2y: 0.2 g, tid) | 24 d | 1. Weight  2. Hb | | 1. T>C*  2. T>C* | NR | NR |  |  |
| Xie 2006 | 54(31:23)  ;2-11y | 42(22:20)  ;2-12y | 1. Hongyi Buxue compound oral solution  (10 mL, tid)  2. (C) | 1. Ferrous sulfate  (4 mg/kg, tid)  2. Vitamin B12, Vitamin E | 8 w | 1. TER  2. RBC  3. Hct  4. Hb  5. SF  6. AE | | 1. T>C*  2. T>C*  3. T>C*  4. T>C*  5. T>C+  6. Not estimable | None | None |  |  |
| Qin 2007 | 77(39:38) | 71(36:35) | 1. Danggui Buxue decoction  (<1y: take 1 pk in 1 day regardless of time and dosage, 1-2y: 1/5 pk, 5 times/d)  2. (C) | 1. Ferrous sulfate granules  (containing iron 10 mg per 5 g(1bag))  (<1y: 5 g, 1-2y: 10 g, tid) | 4 w | 1. TER | | 1. NS | NR | NR |  |  |
| Li 2011 | 31(14:17)  ;11.2 mo | 30(15:15)  ;10.8 mo | 1. Xingpi Yanger granules  (<1y: 2 g, 1-2y: 4 g, bid)  2. (C) | 1. Ferrous sulfate granules (containing 10 mg of iron per 5 g)  (<1y: 5g, 1-2y: 10g, tid) | 12 w | 1. TER  2. Hb  3. SF  4. Weight  5. AE  6. Recurrence rate | | 1. NS  2. NS  3. NS  4. NS  5. NS  6. NS | None | Nausea (2), anorexia (2), diarrhea (1) |  |  |
| Zheng 2011 | 50 | 50 | 1. Yixuesheng capsules (0.25 g/capsule)  (1-3y: 0.25 g, 3-6y: 0.5 g, tid)  2. (C) | 1. Iron dextran oral solution (containing 25 mg of iron per 5 mL [1 bottle])  (<5kg: 5 mL/d, 5-9kg: 10 mL/d, >9kg: 10-20 mL/d, tid) | 4 w | 1. TER  2. Hb  3. RBC  4. MCV  5. MCHC | | 1. T>C+  2. T>C*  3. T>C*  4. T>C*  5. T>C+ | NR | NR |  |  |
|  | 100(56:44)  ;1-6y | |  |  |  |  |  |  |  |  |  |  |
| Zhang 2012 | 35(20:15)  ;2.6 (0.25-7y) | 35(19:16)  ;2.3 (0.16-6y) | 1. Yigong Buxue decoction  2. (C) | 1. Ferrous succinate (100 mg/tablet)  (2 mg/(kg·d), qd or bid, for 1-2 w: daily, for 6 w after Hb value is normal: twice a week)  2. Oral folic acid and vitamin C can be given according to the condition of the child | 8 w | 1. TER  2. AE | | 1. T>C*  2. NS | Nausea, vomiting (1) | Nausea, vomiting, anorexia, abdominal distension and pain (6) |  |  |
| Xiao 2012 | 54(30:24)  ;4.86±2.56 | 54(28:26)  ;4.78±2.68 | 1. Xingpi Yanger granules  (≤3y: 2 g, bid, >3y: 2 g, tid)  2. (C) | 1. Iron dextran oral solution  (5 mg/(kg·d), tid) | 30 d | 1. TER  2. AE | | 1. T>C*  2. T<C* | Epigastric discomfort (1), constipation (1) | Anorexia (7), nausea and vomiting (1), epigastric discomfort (1), diarrhea (1), constipation (1) |  |  |
| Tian 2013 | 48(24:24)  ;16.64±5.15  (0.5-3y) | 47(23:24)  ;16.75±5.34 (0.5-3y) | 1. Xingpi Yanger granules (2 g/bag)  (<1y: 2 g, ≥1y: 4 g, bid  2. (C) | 1. Iron dextran oral solution (containing 25 mg of iron per 5 mL[1bottle])  (5 mg/(kg·d), tid)  2. Dietary guidance (add iron-rich foods, such as pork liver, pig blood, lean meat, dairy products, beans, and fresh fruits, green leafy vegetables) | 3 mo | 1. Hb  2. MCV  3. MCHC  4. AE | | 1. T>C*  2. T>C*  3. T>C*  4. Not estimable | None | None |  |  |
| Lu 2014 | 45(23:22)  ;2.4±0.4  (11mo-5y) | 45(26:19)  ;2.5±0.3  (1-5.5y) | 1. Xingpi Yanger granules  (<1y: 2 g, bid, 1-2y: 4 g, bid, 3-6y: 4 g, tid)  2. (C) | 1. Iron dextran oral solution  (5-9kg: 25 mg, bid, ≥9kg: 25 mg, tid) | 12 w | 1. TER  2. RBC  3. Hb  4. MCV  5. MCH  6. MCHC | | 1. T>C*  2. T>C*  3. T>C*  4. T>C*  5. T>C*  6. T>C* | NR | NR |  |  |
| Qin 2015 | 60(37:23)  ;2.3±0.5  (0.5-1y) | 60(36:24)  ;2.4±0.8 (5mo-11y) | 1. Modified Bazhen decoction  (1 pk, qd)  2. (C) | 1. Ferrous succinate (100 mg/tablet)  (for 1-2 w: 1-2 mg/kg, tid, after Hb is normal, for 6 w: 2 mg/kg, twice a week)  2. Dietary guidance | 30 d | 1. TER  2. Mean healing time  3. AE | | 1. T>C*  2. T<C*  3. T<C* | Mild nausea, vomiting, gastrointestinal discomfort (2) | Gastrointestinal discomfort (9) |  |  |
| Li 2015 | 38(20:18)  ;4.0±0.5 (0.5-7y) | 38(21:17)  ;3.5±0.5  (0.8-6.5y) | 1. Qixue granules (5 g/bag)  (<2y: 2.5 g, tid, 2-7y: 5 g, bid)  2. (C) | 1. Ferrous sulfate (0.3 g/tablet)  (30 mg/(kg·d))  2. Vitamin C (0.1 g/tablet) (100-300 mg/d) | 21 d | 1. RBC  2. Hb  3. SI  4. TIBC  5. TER  6. AE | | 1. T>C*  2. T>C*  3. T>C*  4. T<C*  5. T<C*  6. NS | Vomiting (1) | Vomiting (1), diarrhea (1), headache (2) |  |  |
| Feng 2015 | 66(34:32)  ;20.7±7.4mo  (10-35mo) | 62(35:27)  ;18.3±9.7mo (0.75-3y) | 1. Self-made Buxue decoction  (1/3 pk, 3 tid, every two days) | 1. Ferrous gluconate  (1 mL/(kg·d), tid, every two days) | 8 w | 1. TER  2. RBC  3. Hb  4. SI  5. SF  6. AE | | 1. NS  2. NS  3. T>C*  4. NS  5. T>C*  6. Not estimable | None | None |  |  |
| Gu 2015 | 41 | 41 | 1. Modified Guipi decoction  (1/2 pk, bid)  2. (C) | 1. Compound iron powder (containing ferrous fumarate 30.8 mg, vitamin B1 0.6 mg, vitamin B6 0.6 mg, etc.)  (1.5-2 bags, qd or bid)  2. Vitamin C | 4 w | 1. TER  2. Hb  3. MCH  4. MCHC  5. MCV  6. SI | | 1. T>C*  2. T>C*  3. T>C*  4. T>C*  5. T>C*  6. T>C* | NR | NR |  |  |
| Wu 2017 | 46(21:25)  ;7.4±1.7  (3-12y) | 46(26:20)  ;6.7±1.2  (3-11y) | 1. Jianpi Yiqi Shengxue decoction  (1/2 pk, bid)  2. (C) | 1. Iron dextran solution  (5 mL, tid) | 60 d | 1. SF  2. SI  3. TIBC  4. Hb  5. MCH  6. MCV  7. TER | | 1. T>C*  2. T>C*  3. T<C*  4. T>C*  5. T>C*  6. T>C*  7. T>C* | NR | NR |  |  |
| Ni 2017 | 56 | 56 | 1. Modified Shenling Baizhu powder (3 g/bag)  (<1y: 1 g, 1-3y: 1.5-2 g, 4-6y: 3 g, bid)  2. (C) | 1. Ferrous sulfate  (2 mg/(kg·time), tid)  2.Vitamin C  (50 mg, tid) | 1 mo | 1. TER  2. AE | | 1. T>C*  2. T<C* | Vomiting, abdominal pain, diarrhea (2) | Vomiting, abdominal pain, diarrhea (11) |  |  |
|  | 112(60:52)  ;3.6±1.4 (0.5-6y) | |  |  |  |  |  |  |  |  |  |  |
| Chen 2017 | 21(14:7)  ;5.5±1.0  (1-12y) | 21(12:9)  ;5.7±0.8  (1-12y) | 1. Danggui Buxue decoction  (1/5 pk, 5 times/d)  2. (C) | 1. Iron dextran dispersible tablets  (1-2 mg/kg, tid)  2. Vitamin C  3. Dietary guidance (lean meat, egg yolk, animal offal, fresh vegetables, etc.) | 30 d | 1. TER  2. Mean healing time  3. AE | | 1. T>C*  2. T<C+  3. Not estimable | None | None |  |  |
| Chen 2019 | 35(19:16)  ;3.82±1.60 (0.5-6.5y) | 35(18:17)  ;3.45±1.55 (0.6-6.2y) | 1. Shenling Baizhu powder  (<1y: 1g, 1-3y: 1.5-2 g, 4-6y: 3 g, eod)  2. (C) | 1. Ferrous sulfate  (2 mg/(kg·time), tid)  2. Vitamin C  (50 mg/time, tid) | 4 w | 1. TER  2. Hb  3. MCH  4. MCHC  5. MCV  6. SI  7. AE | | 1. T>C*  2. T>C+  3. T>C+  4. T>C*  5. T>C+  6. T>C+  7. T<C* | Nausea, vomiting (1) | Diarrhea (2), abdominal pain (2), nausea, vomiting (4) |  |  |
| Hao 2020 | 71(42:29)  ;4.31±1.55 (2.5-8.3y) | 72(41:31)  ;4.26±1.67 (2.3-8.1y) | 1. Shengxueling granules (10 g/bag)  (1-3y: 10 g, ≥3y: 15 g, tid)  2. (C) | 1. Iron dextran oral solution (containing 50 mg of iron per 10 mL[1bottle])  (10 mL, qd)  2. Vitamin C | 30 d | 1. TER  2. Disappearance time of clinical symptoms  3. MCHC  4. MCV  5. SI  6. Hb  7. AE | | 1. T>C*  2. T<C*  3. T>C*  4. T>C*  5. T>C*  6. T>C*  7. NS | Constipation (3), nausea and vomiting (3), diarrhea (2), oral odor (3) | Constipation (2), nausea and vomiting (3), diarrhea (2), oral odor (2) |  |  |
| Deng 2020 | 60(30:30)  ;2.9±1.1 (0.6-6y) | 60(32:28)  ;3.0±1.5 (0.5-5y) | 1. Yiqi Jianpi Bushen Shengxue decoction  (1/2 pk, bid)  2. (C) | 1. Iron dextran oral solution  (5 mg/(kg·d), tid) | 4 w | 1. TER  2. Hb  3. RBC  4. MCV  5. MCHC  6. Total renal blood flow  7. SI  8. AE | | 1. T>C*  2. T>C*  3. T>C*  4. T>C*  5. T>C*  6. T>C*  7. T>C*  8. NS | Nausea and vomiting (1), abdominal pain and diarrhea (1) | Nausea and vomiting (2), abdominal pain and diarrhea (1) |  |  |
| Li 2021 | 60(34:26)  ;4.79±1.23 (0.5-8y) | 60(32:28)  ;4.83±1.25 (0.5-9y) | 1. Guipi pill (9 g/pill)  (1 pill, tid)  2. (C) | 1. Compound ferrous sulfate granules (containing ferrous sulfate 50 mg, vitamin C 30 mg)  (1 bag, qd, pc)  2. Dietary supplement (foods rich in iron and vitamin C) | 4 w | 1. TCM syndrome score  2. SI  3. SF  4. TIBC  5. AE | | 1. T<C+  2. NS  3. NS  4. NS  5. NS | Nausea (3), indigestion (2) | Nausea (2), indigestion (1) |  |  |
| Zhang 2021 | 43(28:15)  ;7.21±1.87  (3-13y) | 43(26:17)  ;7.05±1.72 (3-13y) | 1. Shengxuebao granules  (8 g, bid)  2. (C) | 1. Ferrous lactate tablet  (0.1 g, tid) | 8 w | 1. TER  2. WHOQOL-bref  3. RBC  4. Hb  5. Reticulocyte  6. SF  7. TS  8. AE | | 1. T>C*  2. T>C*  3. T>C*  4. T>C*  5. T>C*  6. T>C*  7. T>C*  8. NS | Nausea (3), vomiting (1), constipation (1), epigastric abdominal pain (1) | Nausea (1), vomiting (3), constipation (2), epigastric abdominal pain (2) |  |  |
| Chen 2023 | 38(22:16)  ;3.36±1.05  (1-6y) | 38(20:18)  ;3.72±1.30 (1-7y) | 1. Jianpi Yiqi Shengxue decoction  (1/3 pk, tid)  2. (C) | 1. Iron protein succinylate oral solution (containing ferric 40 mg per 15 mL[1bottle])  (1.5 mL/(kg·d), bid) | 1 mo | 1. TCM syndrome score  2. TER  3. Hb  4. RBC  5. SI  6. SF  7. TIBC  8. MCV  9. MCHC | | 1. NS  2. T>C*  3. T>C*  4. T>C*  5. T>C*  6. T>C*  7. T<C*  8. T>C*  9. T>C* | NR | NR |  |  |
| Qian 2023 | 40(25:15)  ;4.23±1.34 | 40(23:17)  ;4.42±1.52 | 1. Yun-Pi Buxue decoction  (1/2 pk, bid)  2. (C) | 1. Iron dextran oral solution  (10 mL, qd, pc)  2. Vitamin C  (0.1 g, qd, pc) | 30 d | 1. TER  2. Hb  3. SI  4. TS  5. MCV  6. MCHC  7. Recurrence rate  8. AE | | 1. T>C*  2. T>C*  3. T>C*  4.-T>C*  5. T>C*  6. T>C*  7. T<C*  8. NS | Nausea and vomiting (2), diarrhea (2), constipation (1), oral odor (2) | Nausea and vomiting (2), diarrhea (1), constipation (1), oral odor (1) |  |  |

Notes: *, *P* < 0.05; +, *P* < 0.01; NS, *P* > 0.05; AE, adverse event; bid, twice a day; C, control group; d, day; eod, every other day; F, female; Hb, hemoglobin; Hct, hematocrit; y, year; NR, not reported; M, male; mo, month; MCV, mean corpuscular volume; MCH, mean corpuscular hemoglobin; MCHC, mean corpuscular hemoglobin concentration; pc, post cibum; pk, pack; qd, once a day; qid, four times a day; RBC, red blood cell; RCT, randomized controlled trial; SF, serum ferritin; SI, serum iron; T, treatment group; TCM, traditional Chinese medicine; TER, total effective rate; TIBC, total iron binding capacity; tid, three times a day; TS, transferrin saturation; w, week; WHOQOL-BREF, Korean WHO quality of life scale abbreviated version.

# Supplementary Table 3. East Asian herbal medicine used in the treatment group

| **Study ID** | **Prescription name (Chinese name)** | **Composition**  **(Accepted name [Family; Latin name])** | **Type of preparation** | **Extraction procedure/Product (Company; Approval number)** |
| --- | --- | --- | --- | --- |
| She 2001 | Modified Shenling Baizhu powder | *Astragalus mongholicus Bunge [Fabaceae; Astragali Radix]*, *Hordeum vulgare L. [Poaceae; Hordei Fructus Germinatus], Coix lacryma-jobi var. ma-yuen (Rom.Caill.) Stapf [Poaceae; Coicis Semen]* 15 g, *Codonopsis pilosula subsp. tangshen (Oliv.) D.Y.Hong [Campanulaceae; Codonopsis Pilosulae Radix]*, *Atractylodes macrocephala Koidz. [Asteraceae; Atractylodis Rhizoma Alba]*, *Lablab purpureus subsp. purpureus [Fabaceae; Dolichoris Semen]* 12 g, *Wurfbainia villosa (Lour.) Škorničk. & A.D.Poulsen [Zingiberaceae; Amomi Fructus]* 3 g | Powder | Boiled in water |
| Li 2003 | Xingpi Yanger granules | *Gerbera piloselloides (L.) Cass. [Asteraceae; Gerbera piloselloides]*, *Emilia sonchifolia (L.) DC. [Asteraceae; Emilia sonchifolia]*, *Nardostachys jatamansi (D.Don) DC. [Caprifoliaceae; Valeriana jatamansi]* | Granules | Guizhou Jianxing Pharmaceutical Co., Ltd.; National Medicine Standard Z20025415 |
| Sun 2004 | Modified Danggui Buxue decoction | *Astragalus mongholicus Bunge [Fabaceae; Astragali Radix]* 500 g, *Lycium barbarum L. [Solanaceae; Lycii Fructus]*, *Ziziphus jujuba Mill. [Rhamnaceae; Zizyphi Fructus]* 300 g, *Angelica sinensis (Oliv.) Diels [Apiaceae; Angelicae Sinensis Radix]*, *Wolfiporia extensa (Peck) Ginns [Polyporaceae; Poria Sclerotium]*, *Atractylodes macrocephala Koidz. [Asteraceae; Atractylodis Rhizoma Alba]*, *Dioscorea polystachya Turcz. [Dioscoreaceae; Dioscorea Rhizome],* *Reynoutria multiflora (Thunb.) Moldenke [Polygonaceae; Polygoni Multiflori Radix]* 240 g, *Equus asinus L [Equidae; Asini Corii Colla]* 150 g, *Aucklandia costus Falc. [Asteraceae; Aucklandiae Radix]*, *Glycyrrhiza glabra L. [Fabaceae; Glycyrrhizae Radix et Rhizoma]* 100 g | Decoction | Boiled in water |
| Xie 2006 | Hongyi Buxue compound oral solution | *Arachis hypogaea L. [Fabaceae; Arachis hypogaea (Pericarpium)]*, *Lycium barbarum L. [Solanaceae; Lycii Fructus]*, *Ziziphus jujuba Mill. [Rhamnaceae; Zizyphi Fructus]*, *Auricularia auricula-judae (Bull.) Quél. [Auriculariaceae; Auricularia auricula]* | Solution | Xiangyu Pharmaceutical Industry Co., Ltd. |
| Qin 2007 | Danggui Buxue decoction | *Astragalus mongholicus Bunge [Fabaceae; Astragali Radix]* 5–15 g, *Atractylodes macrocephala Koidz. [Asteraceae; Atractylodis Rhizoma Alba]*, *Wolfiporia extensa (Peck) Ginns [Polyporaceae; Poria Sclerotium]*, *Citrus reticulata Blanco [Rutaceae; Citri Unshius Pericarpium]*, *Gallus gallus domesticus Brisson [Phasianidae; Galli Stomachichum Corium]* 9–10 g, *Angelica sinensis (Oliv.) Diels [Apiaceae; Angelicae Sinensis Radix]* 3–9 g, *Glycyrrhiza glabra L. [Fabaceae; Glycyrrhizae Radix et Rhizoma]* 3–5 g | Decoction | Boiled in water |
| Shao 2010 | Qixue granules | *Astragalus mongholicus Bunge [Fabaceae; Astragali Radix]*, *Ziziphus jujuba Mill. [Rhamnaceae; Zizyphi Fructus]*, Blood meal | Granules | Xi'an Jiaotong University Ruixin Pharmaceutical Co., Ltd. |
| Li 2011 | Xingpi Yanger granules | *Gerbera piloselloides (L.) Cass. [Asteraceae; Gerbera piloselloides]*, *Emilia sonchifolia (L.) DC. [Asteraceae; Emilia sonchifolia]*, *Nardostachys jatamansi (D.Don) DC. [Caprifoliaceae; Valeriana jatamansi]*, *Pittosporum glabratum Lindl. [Pittosporaceae; Pittosporum illicioides Makino]* | Granules | Guizhou Jianxing Pharmaceutical Co., Ltd; National Medicine Standard Z20025415 |
| Zheng 2011 | Yixuesheng capsules | *Equus asinus L [Equidae; Asini Corii Colla], Cervus nippon Temminck [Cervidae; Cervi Cornus Colla (deer antler glue)], Pelodiscus sinensis (Wiegmann) [Trinoychidae; Pelodiscis Carapax], Rehmannia glutinosa (Gaertn.) DC. [Orobanchaceae; Rehmanniae Radix Preparata], Paeonia lactiflora Pall. [Paeoniaceae; Radix Paeoniae Alba], Angelica sinensis (Oliv.) Diels [Apiaceae; Angelicae Sinensis Radix], Achyranthes bidentata Blume [Amaranthaceae; Achyranthis Radix], Hominis Placenta, Codonopsis pilosula subsp. tangshen (Oliv.) D.Y.Hong [Campanulaceae; Codonopsis Pilosulae Radix], Astragalus mongholicus Bunge [Fabaceae; Astragali Radix], Atractylodes macrocephala Koidz. [Asteraceae; Atractylodis Rhizoma Alba]* | Capsule | Jilin Jinfukang Pharmaceutical Co., Ltd.; National Medicine Standard Z19983056 |
| Sun 2011 | Qixue granules | *Angelica sinensis (Oliv.) Diels [Apiaceae; Angelicae Sinensis Radix], Astragalus mongholicus Bunge [Fabaceae; Astragali Radix], Ziziphus jujuba Mill. [Rhamnaceae; Zizyphi Fructus], Citrus reticulata Blanco [Rutaceae; Citri Unshius Pericarpium], Crataegus laevigata (Poir.) DC. [Rosaceae; Crataegi Fructus]*, Blood meal | Granules | Not reported |
| Zhou 2011 | Qixue granules | *Angelica sinensis (Oliv.) Diels [Apiaceae; Angelicae Sinensis Radix], Astragalus mongholicus Bunge [Fabaceae; Astragali Radix], Ziziphus jujuba Mill. [Rhamnaceae; Zizyphi Fructus], Citrus reticulata Blanco [Rutaceae; Citri Unshius Pericarpium], Crataegus laevigata (Poir.) DC. [Rosaceae; Crataegi Fructus]*, Blood meal | Granules | Xi'an Jiaotong University Ruixin Pharmaceutical Co., Ltd. |
| Zhang 2012 | Yigong Buxue decoction | *Astragalus mongholicus Bunge [Fabaceae; Astragali Radix]* 5–15 g, *Codonopsis pilosula subsp. tangshen (Oliv.) D.Y.Hong [Campanulaceae; Codonopsis Pilosulae Radix]* 10 g, *Angelica sinensis (Oliv.) Diels [Apiaceae; Angelicae Sinensis Radix]* 3–9 g, *Atractylodes macrocephala Koidz. [Asteraceae; Atractylodis Rhizoma Alba], Wolfiporia extensa (Peck) Ginns [Polyporaceae; Poria Sclerotium], Atractylodes lancea (Thunb.) DC. [Asteraceae; Atractylodis Rhizoma], Citrus reticulata Blanco [Rutaceae; Citri Unshius Pericarpium], Gallus gallus domesticus Brisson [Phasianidae; Galli Stomachichum Corium]* 6–10 g, *Glycyrrhiza glabra L. [Fabaceae; Glycyrrhizae Radix et Rhizoma]* 3–5 g | Decoction | Boiled in water |
| Xiao 2012 | Xingpi Yanger granules | *Astragalus mongholicus Bunge [Fabaceae; Astragali Radix], Gerbera piloselloides (L.) Cass. [Asteraceae; Gerbera piloselloides], Emilia sonchifolia (L.) DC. [Asteraceae; Emilia sonchifolia], Nardostachys jatamansi (D.Don) DC. [Caprifoliaceae; Valeriana jatamansi]*. | Granules | Guizhou Jianxing Pharmaceutical Co., Ltd. |
| Tian 2013 | Xingpi Yanger granules | *Gerbera piloselloides (L.) Cass. [Asteraceae; Gerbera piloselloides], Emilia sonchifolia (L.) DC. [Asteraceae; Emilia sonchifolia], Pittosporum glabratum Lindl. [Pittosporaceae; Pittosporum illicioides Makino], Nardostachys jatamansi (D.Don) DC. [Caprifoliaceae; Valeriana jatamansi]* | Granules | Guizhou Jianxing Pharmaceutical Co., Ltd.; National Medicine Standard Z20025415 |
| Lu 2014 | Xingpi Yanger granules | *Emilia sonchifolia (L.) DC. [Asteraceae; Emilia sonchifolia], Gerbera piloselloides (L.) Cass. [Asteraceae; Gerbera piloselloides], Pittosporum glabratum Lindl. [Pittosporaceae; Pittosporum illicioides Makino], Nardostachys jatamansi (D.Don) DC. [Caprifoliaceae; Valeriana jatamansi]* | Granules | Not reported |
| Li 2015 | Qixue granules | *Angelica sinensis (Oliv.) Diels [Apiaceae; Angelicae Sinensis Radix], Astragalus mongholicus Bunge [Fabaceae; Astragali Radix], Ziziphus jujuba Mill. [Rhamnaceae; Zizyphi Fructus], Citrus reticulata Blanco [Rutaceae; Citri Unshius Pericarpium], Crataegus laevigata (Poir.) DC. [Rosaceae; Crataegi Fructus]*, Blood meal | Granules | Xi'an Jiaotong University Ruixin Pharmaceutical Co., Ltd.; National Drug Approval Number B20020961 |
| Gu 2015 | Modified Guipi decoction | *Astragalus mongholicus Bunge [Fabaceae; Astragali Radix]* 12 g, *Codonopsis pilosula subsp. tangshen (Oliv.) D.Y.Hong [Campanulaceae; Codonopsis Pilosulae Radix], Atractylodes macrocephala Koidz. [Asteraceae; Atractylodis Rhizoma Alba], Angelica sinensis (Oliv.) Diels [Apiaceae; Angelicae Sinensis Radix], Wolfiporia extensa (Peck) Ginns [Polyporaceae; Poria Sclerotium], Dimocarpus longan Lour. [Sapindaceae; Longan Arillus], Ziziphus jujuba Mill. [Rhamnaceae; Zizyphi Semen], Equus asinus L [Equidae; Asini Corii Colla]* 10 g, *Polygala tenuifolia Willd. [Polygalaceae; Polygalae Radix], Citrus reticulata Blanco [Rutaceae; Citri Unshius Pericarpium], Glycyrrhiza glabra L. [Fabaceae; Glycyrrhizae Radix et Rhizoma]* 6 g, *Ziziphus jujuba Mill. [Rhamnaceae; Zizyphi Fructus]* 6 pieces, *Zingiber officinale Roscoe [Zingiberaceae; Rhizoma Crudus]* 2 slices | Decoction | Boiled in water |
| Qin 2015 | Modified Bazhen decoction | *Astragalus mongholicus Bunge [Fabaceae; Astragali Radix]* 12 g, *Codonopsis pilosula subsp. tangshen (Oliv.) D.Y.Hong [Campanulaceae; Codonopsis Pilosulae Radix], Rehmannia glutinosa (Gaertn.) DC. [Orobanchaceae; Rehmanniae Radix Preparata]* 10 g, *Angelica sinensis (Oliv.) Diels [Apiaceae; Angelicae Sinensis Radix], Wolfiporia extensa (Peck) Ginns [Polyporaceae; Poria Sclerotium], Atractylodes macrocephala Koidz. [Asteraceae; Atractylodis Rhizoma Alba]* 9 g, *Paeonia lactiflora Pall. [Paeoniaceae; Radix Paeoniae Alba]* 6 g, *Conioselinum anthriscoides ‘Chuanxiong’ [Apiaceae; Cnidii Rhizoma]* 5 g, *Glycyrrhiza glabra L. [Fabaceae; Glycyrrhizae Radix et Rhizoma]* 3 g, *Ziziphus jujuba Mill. [Rhamnaceae; Zizyphi Fructus]* 2 slices | Decoction | Boiled in water |
| Feng 2015 | Self-made Buxue formula | *Astragalus mongholicus Bunge [Fabaceae; Astragali Radix], Angelica sinensis (Oliv.) Diels [Apiaceae; Angelicae Sinensis Radix]* 6 g, *Dioscorea polystachya Turcz. [Dioscoreaceae; Dioscorea Rhizome] Crataegus laevigata (Poir.) DC. [Rosaceae; Crataegi Fructus], Prunus mume (Siebold) Siebold & Zucc. [Rosaceae; Mume Fructus], Hippophae rhamnoides L. [Elaeagnaceae; Sea buckthorn], Ziziphus jujuba Mill. [Rhamnaceae; Zizyphi Fructus]* 3 g | Not reported | Boiled in water |
| Wu 2017 | Jianpi Yiqi Shengxue decoction | *Astragalus mongholicus Bunge [Fabaceae; Astragali Radix], Codonopsis pilosula subsp. tangshen (Oliv.) D.Y.Hong [Campanulaceae; Codonopsis Pilosulae Radix], Spatholobus suberectus Dunn [Fabaceae; Spatholobi Caulis], Atractylodes macrocephala Koidz. [Asteraceae; Atractylodis Rhizoma Alba], Wolfiporia extensa (Peck) Ginns [Polyporaceae; Poria Sclerotium], Reynoutria multiflora (Thunb.) Moldenke [Polygonaceae; Polygoni Multiflori Radix]* 10 g, *Gallus gallus domesticus Brisson [Phasianidae; Galli Stomachichum Corium]* 8 g, *Ligustrum lucidum W.T.Aiton [Oleaceae; Ligustri Fructus]* 6 g, *Citrus reticulata Blanco [Rutaceae; Citri Unshius Pericarpium]* 3 g | Decoction | Boiled in water |
| Ni 2017 | Modified Shenling Baizhu powder | *Pseudostellaria heterophylla (Miq.) Pax [Caryophyllaceae; Pseudostellariae Radix]*, *Atractylodes macrocephala Koidz. [Asteraceae; Atractylodis Rhizoma Alba]*, *Dioscorea polystachya Turcz. [Dioscoreaceae; Dioscorea Rhizome]* *Crataegus laevigata (Poir.) DC. [Rosaceae; Crataegi Fructus]*, *Hordeum vulgare L. [Poaceae; Hordei Fructus Germinatus]*, *Coix lacryma-jobi var. ma-yuen (Rom.Caill.) Stapf [Poaceae; Coicis Semen]*, *Wolfiporia extensa (Peck) Ginns [Polyporaceae; Poria Sclerotium]*, *Lablab purpureus subsp. purpureus [Fabaceae; Dolichoris Semen]*, *Euryale ferox Salisb. [Nymphaeaceae; Euryalis Semen]*, *Nelumbo nucifera Gaertn. [Nelumbonaceae; Nelumbinis Semen]* 6 g, *Citrus reticulata Blanco [Rutaceae; Citri Unshius Pericarpium], Acorus gramineus Aiton [Acoraceae; Acori graminei Rhizoma], Glycyrrhiza glabra L. [Fabaceae; Glycyrrhizae Radix et Rhizoma]* 3 g | Powder | According to this ratio, make a powder and drink it as a dilute |
| Chen 2017 | Danggui Buxue decoction | *Astragalus mongholicus Bunge [Fabaceae; Astragali Radix]* 5–15 g, *Angelica sinensis (Oliv.) Diels [Apiaceae; Angelicae Sinensis Radix]* 3–9 g, *Gallus gallus domesticus Brisson [Phasianidae; Galli Stomachichum Corium], Wolfiporia extensa (Peck) Ginns [Polyporaceae; Poria Sclerotium], Atractylodes macrocephala Koidz. [Asteraceae; Atractylodis Rhizoma Alba], Citrus reticulata Blanco [Rutaceae; Citri Unshius Pericarpium]* 9–10 g, *Glycyrrhiza glabra L. [Fabaceae; Glycyrrhizae Radix et Rhizoma]* 3–5 g | Decoction | Boiled in water |
| Chen 2019 | Shenling Baizhu powder | *Lablab purpureus subsp. purpureus [Fabaceae; Dolichoris Semen], Pseudostellaria heterophylla (Miq.) Pax [Caryophyllaceae; Pseudostellariae Radix], Crataegus laevigata (Poir.) DC. [Rosaceae; Crataegi Fructus], Euryale ferox Salisb. [Nymphaeaceae; Euryalis Semen], Hordeum vulgare L. [Poaceae; Hordei Fructus Germinatus], Dioscorea polystachya Turcz. [Dioscoreaceae; Dioscorea Rhizome] Nelumbo nucifera Gaertn. [Nelumbonaceae; Nelumbinis Semen], Coix lacryma-jobi var. ma-yuen (Rom.Caill.) Stapf [Poaceae; Coicis Semen], Atractylodes macrocephala Koidz. [Asteraceae; Atractylodis Rhizoma Alba]* 6 g, *Citrus reticulata Blanco [Rutaceae; Citri Unshius Pericarpium], Acorus gramineus Aiton [Acoraceae; Acori graminei Rhizoma], Glycyrrhiza glabra L. [Fabaceae; Glycyrrhizae Radix et Rhizoma]* 3 g | Powder | According to this ratio, make a powder and take it with warm water |
| Hao 2020 | Shengxueling granules | *Crataegus laevigata (Poir.) DC. [Rosaceae; Crataegi Fructus], Astragalus mongholicus Bunge [Fabaceae; Astragali Radix], Equus asinus L [Equidae; Asini Corii Colla], Melanteritum, Ziziphus jujuba Mill. [Rhamnaceae; Zizyphi Fructus]* | Granules | Shandong Hongjitang Pharmaceutical Group Co., Ltd.; Batch number 20161231 |
| Deng 2020 | Yiqi Jianpi Bushen Shengxue decoction | *Hordeum vulgare L. [Poaceae; Hordei Fructus Germinatus]* 5–15 g, *Astragalus mongholicus Bunge [Fabaceae; Astragali Radix], Rehmannia glutinosa (Gaertn.) DC. [Orobanchaceae; Rehmanniae Radix Preparata], Dioscorea polystachya Turcz. [Dioscoreaceae; Dioscorea Rhizome]* 3–10 g, *Reynoutria multiflora (Thunb.) Moldenke [Polygonaceae; Polygoni Multiflori Radix]* 3–9 g, *Codonopsis pilosula subsp. tangshen (Oliv.) D.Y.Hong [Campanulaceae; Codonopsis Pilosulae Radix], Conioselinum anthriscoides ‘Chuanxiong’ [Apiaceae; Cnidii Rhizoma], Crataegus laevigata (Poir.) DC. [Rosaceae; Crataegi Fructus], Cuscuta chinensis Lam. [Convolvulaceae; Cuscutae Semen]*, *Ligustrum lucidum W.T.Aiton [Oleaceae; Ligustri Fructus]* 3–5 g, *Atractylodes macrocephala Koidz. [Asteraceae; Atractylodis Rhizoma Alba]* 2–5 g, *Wolfiporia extensa (Peck) Ginns [Polyporaceae; Poria Sclerotium]* 2–6 g, *Equus asinus L [Equidae; Asini Corii Colla], Angelica sinensis (Oliv.) Diels [Apiaceae; Angelicae Sinensis Radix], Glycyrrhiza glabra L. [Fabaceae; Glycyrrhizae Radix et Rhizoma], Citrus reticulata Blanco [Rutaceae; Citri Unshius Pericarpium]* 2–5 g | Decoction | Boiled in water |
| Zhang 2021 | Shengxuebao granules | *Reynoutria multiflora (Thunb.) Moldenke [Polygonaceae; Polygoni Multiflori Radix], Astragalus mongholicus Bunge [Fabaceae; Astragali Radix], Cibotium barometz (L.) J.Sm. [Cyatheaceae; Cibotii Rhizoma], Eclipta prostrata (L.) L. [Asteraceae; Ecliptae Herba], Morus alba L. [Moraceae; Mori Fructus], Ligustrum lucidum W.T.Aiton [Oleaceae; Ligustri Fructus], Paeonia lactiflora Pall. [Paeoniaceae; Radix Paeoniae Alba]* | Granules | Hunan Kangshou Pharmaceutical Co., Ltd.; Batch number 20190721, 20200419 |
| Li 2021 | Guipi pill | *Codonopsis pilosula subsp. tangshen (Oliv.) D.Y.Hong [Campanulaceae; Codonopsis Pilosulae Radix], Atractylodes macrocephala Koidz. [Asteraceae; Atractylodis Rhizoma Alba], Astragalus mongholicus Bunge [Fabaceae; Astragali Radix], Wolfiporia extensa (Peck) Ginns [Polyporaceae; Poria Sclerotium], Polygala tenuifolia Willd. [Polygalaceae; Polygalae Radix], Dimocarpus longan Lour. [Sapindaceae; Longan Arillus], Angelica sinensis (Oliv.) Diels [Apiaceae; Angelicae Sinensis Radix], Aucklandia costus Falc. [Asteraceae; Aucklandiae Radix], Ziziphus jujuba Mill. [Rhamnaceae; Zizyphi Semen], Ziziphus jujuba Mill. [Rhamnaceae; Zizyphi Fructus]* | Pill | Guangdong Jiatai Pharmaceutical Co., Ltd.; National Drug Approval Number Z20025939 |
| Chen 2023 | Jianpi Yiqi Shengxue decoction | *Codonopsis pilosula subsp. tangshen (Oliv.) D.Y.Hong [Campanulaceae; Codonopsis Pilosulae Radix], Atractylodes macrocephala Koidz. [Asteraceae; Atractylodes Rhizome Alba], Astragalus mongholicus Bunge [Fabaceae; Astragali Radix], Spatholobus suberectus Dunn [Fabaceae; Spatholobi Caulis], Reynoutria multiflora (Thunb.) Moldenke [Polygonaceae; Polygoni Multiflori Radix], Wolfiporia extensa (Peck) Ginns [Polyporaceae; Poria Sclerotium]* 10 g, *Ligustrum lucidum W.T.Aiton [Oleaceae; Ligustri Fructus]* 6 g, *Citrus reticulata Blanco [Rutaceae; Citri Unshius Pericarpium]* 3 g | Decoction | Boiled in water |
| Qian 2023 | Yun-Pi Buxue decoction | *Astragalus mongholicus Bunge [Fabaceae; Astragali Radix], Angelica sinensis (Oliv.) Diels [Apiaceae; Angelicae Sinensis Radix], Atractylodes macrocephala Koidz. [Asteraceae; Atractylodis Rhizoma Alba], Wolfiporia extensa (Peck) Ginns [Polyporaceae; Poria Sclerotium], Atractylodes lancea (Thunb.) DC. [Asteraceae; Atractylodis Rhizoma]* 10 g, *Citrus reticulata Blanco [Rutaceae; Citri Unshius Pericarpium]* 6 g | Decoction | Boiled in water |

* Accepted names were based on the Kew’s Medicinal Plant Names Services (MPNS) (https://mpns.science.kew.org/mpns-portal/ accessed 5 Feb 2024).

# Supplementary Table 4. Summary of findings of meta-analysis

| Outcomes | Relative effect  (95% CI) | Anticipated absolute effects (95% CI) | | Number of participants  (studies) | Certainty of the evidence (GRADE) |
| --- | --- | --- | --- | --- | --- |
|  |  | Risk with control | Risk with HM |  |  |
| HM versus oral iron | | | | | |
| Hemoglobin |  |  | MD 11.33 higher  (6.2 higher to 16.46 higher) | 468  (4 RCTs) | ⨁⨁⨁◯  Moderate^a^ |
| Serum ferritin |  |  | SMD 0.71 SD higher  (2.14 lower to 3.57 higher) | 180  (2RCTs) | ⨁◯◯◯  Very low^a,b,c^ |
| TER | RR 1.12  (1.02 to 1.23) | 856 per 1,000 | 959 per 1,000  (874 to 1,000) | 503  (4RCTs) | ⨁⨁⨁◯  Moderate^a^ |
| RBC |  |  | MD 0.22 higher  (0.04 lower to 0.48 higher) | 468  (4RCTs) | ⨁⨁⨁◯  Moderate^a^ |
| Serum iron |  |  | MD 3.53 higher  (0.41 lower to 7.48 higher) | 288  (2RCTs) | ⨁◯◯◯  Very low^a,b,c^ |
| TIBC |  |  | MD 11.33 lower  (17.31 lower to 5.36 lower) | 388  (3RCTs) | ⨁⨁◯◯  Low^a,c^ |
| Incidence of AEs | RR 0.08  (0.01 to 0.56) | 92 per 1,000 | 7 per 1,000  (1 to 52) | 262  (3RCTs) | ⨁⨁⨁◯  Moderate^a^ |
| HM plus oral iron versus oral iron | | | | | |
| Hemoglobin |  |  | MD 13.1 higher  (10.47 higher to 15.73 higher) | 1615  (16RCTs) | ⨁◯◯◯  Very low^a,b,e^ |
| Serum ferritin |  |  | SMD 1.54 SD higher  (0.01 higher to 3.07 higher) | 659  (7RCTs) | ⨁⨁◯◯  Low^a,b^ |
| TER | RR 1.14  (1.09 to 1.19) | 831 per 1,000 | 947 per 1,000  (905 to 988) | 1900  (20RCTs) | ⨁⨁◯◯  Low^a,d^ |
| RBC |  |  | MD 0.48 higher  (0.29 higher to 0.67 higher) | 772  (8RCTs) | ⨁⨁◯◯  Low^a,b^ |
| Serum iron |  |  | MD 4.34 higher  (2.64 higher to 5.74 higher) | 987  (10RCTs) | ⨁◯◯◯  Very low^a,b,d^ |
| TIBC |  |  | MD 5.87 lower  (10.44 lower to 1.29 lower) | 364  (4RCTs) | ⨁◯◯◯  Very low^a,c,e,f^ |
| Incidence of AEs | RR 0.49  (0.34 to 0.70) | 109 per 1,000 | 53 per 1,000  (37 to 76) | 1519  (16RCTs) | ⨁◯◯◯  Very low^a,d,e^ |
| MCH |  |  | MD 3.62 higher  (2.52 higher to 4.72 higher) | 334  (4RCTs) | ⨁⨁◯◯  Low^a,c^ |
| MCV |  |  | MD 8.18 higher  (5.83 higher to 10.52 higher) | 948  (10RCTs) | ⨁◯◯◯  Very low^a,b,d^ |
| Time to recovery (day) |  |  | MD 9.39 lower  (14.95 lower to 3.82 lower) | 305  (3RCTs) | ⨁◯◯◯  Very low^a,b,c^ |

Notes: GRADE, grading of recommendations assessment, development, and evaluation; HM, herbal medicine; RCT, randomized controlled trial; TER, total effective rate; CI, confidence interval; MD, mean difference; RR, risk ratio; SMD, standardized mean difference; AEs, adverse events; RBC, red blood cell; TIBC, total iron binding capacity; MCH, mean corpuscular hemoglobin; MCV, mean corpuscular volume.

a. Unclear risk of selection, performance, and detection bias.

b. I2 ≥ 75% with same direction of the effect size.

c. Sample size (continuous variables) < 400

d. Funnel plots appear to have asymmetry.

e. The 95% confidence interval overlapped too broad spectrum.
